# Detecting the corruption of online questionnaires by artificial intelligence

**DOI:** 10.3389/frobt.2023.1277635

**Published:** 2024-02-02

**Authors:** Benjamin Lebrun, Sharon Temtsin, Andrew Vonasch, Christoph Bartneck

**Affiliations:** ^1^ School of Psychology, Speech, and Hearing, University of Canterbury, Christchurch, New Zealand; ^2^ Department of Computer Science and Software Engineering, University of Canterbury, Christchurch, New Zealand

**Keywords:** AI, detection, data quality, imitation game, large language models, online questionnaires, reliability

## Abstract

Online questionnaires that use crowdsourcing platforms to recruit participants have become commonplace, due to their ease of use and low costs. Artificial intelligence (AI)-based large language models (LLMs) have made it easy for bad actors to automatically fill in online forms, including generating meaningful text for open-ended tasks. These technological advances threaten the data quality for studies that use online questionnaires. This study tested whether text generated by an AI for the purpose of an online study can be detected by both humans and automatic AI detection systems. While humans were able to correctly identify the authorship of such text above chance level (76% accuracy), their performance was still below what would be required to ensure satisfactory data quality. Researchers currently have to rely on a lack of interest among bad actors to successfully use open-ended responses as a useful tool for ensuring data quality. Automatic AI detection systems are currently completely unusable. If AI submissions of responses become too prevalent, then the costs associated with detecting fraudulent submissions will outweigh the benefits of online questionnaires. Individual attention checks will no longer be a sufficient tool to ensure good data quality. This problem can only be systematically addressed by crowdsourcing platforms. They cannot rely on automatic AI detection systems and it is unclear how they can ensure data quality for their paying clients.

## 1 Introduction

The use of crowdsourcing platforms to recruit participants for online questionnaires has always been susceptible to abuse. Bad actors could randomly click answers to quickly earn money, even at scale. Until recently, a solution to this problem was to ask online participants to complete open-ended responses that could not be provided through random button-clicking. However, the development of large language models, such as ChatGPT or Bard, threatens the viability of this solution. This threat to data quality has to be understood in the wider context of methodological challenges that all add up to what is now famously termed the “replication crisis”.

The replication crisis, initially observed in the field of psychology and human behavior, has also been shown to occur in other domains, including computer science, chemistry, biology, and medicine (Peng, 2011; Baker, 2016). The crisis is based on the difficulty of replicating the results of previous studies. A 2015 Open Science study attempted to replicate 100 psychology studies. In this study, 97% of the studies showed significant results but the authors only succeeded in replicating 36% of them (Open Science Collaboration, 2015). Human–Robot Interaction (HRI) is a multidisciplinary field and is no exception to this crisis (Irfan et al., 2018; Leichtmann et al., 2022; Leichtmann and Nitsch, 2020a; Leichtmann and Nitsch, 2020b; Strait et al., 2020). Ullman et al. (2021) attempted to replicate their own underpowered HRI study (Ullman and Malle, 2017) using different replication methods (i.e., conceptual, direct, and online). Each of these attempts, while having a more than acceptable sample size and power, did not replicate the results of the original study. It seems that the lack of power in the original study prevented the results from being reproduced and that the previously observed significant effect probably does not exist, but represents a Type I error (false positive).

There are several factors that contribute to the replication crisis. Some are specific to HRI, while others apply to all fields of study. Several studies have investigated the factors that may contribute to replication difficulties in general, such as sample size, power, recruitment methods, and publication bias (Peng, 2011; Baxter et al., 2016; Swiatkowski and Dompnier, 2017; Leichtmann and Nitsch, 2020a; Belpaeme, 2020). A Nature survey (Baker, 2016) asked 1,576 researchers the reasons that might contribute to the replication crisis and most of them cited the pressure to publish, but also biases in methods and statistical analyses. For example, researchers may conduct and report statistical analyses that inappropriately increase the odds of finding significant results (Kerr, 1998; Simmons et al., 2011).

Baxter et al. (2016) analyzed papers presented at HRI conferences and reported that most of the sample sizes were below 20 participants per condition. This small sample size per condition might lead to under-powered and less sensitive studies, as participants are not used as their own controls and individual differences might influence results and their interpretation. In this context, only large effect sizes might be detected. For these reasons, it is important to check the required sample size *a priori* using expected power, alpha, and effect size. The sample size might therefore depend on the design of the study (a within-participants design would require fewer participants than a between-participants design) and the expected effect size. Leichtmann and Nitsch (2020a) argue that replication difficulties are based on a lack of theory and transparency, and the use of methodologies that are not powerful enough. They suggest increasing sample sizes and pre-registering studies. Furthermore, computer code should be made available (Peng, 2011). Following these recommendations, researchers are increasingly making their materials and code available, pre-registering their studies, and increasing their sample sizes (Tenney et al., 2021). However, in an effort to increase sample sizes, researchers are increasingly relying on online data collection, rather than in-person studies, which comes with trade-offs (Baumeister, 2016).

Ullman et al. (2021) and Strait et al. (2020) argue that the difficulty of replicating results in the field of HRI is due to the wide variety of robots used. Robots used in HRI are often expensive and some robots have only ever been built in small numbers. Such specialist robots can be complicated to use (Leichtmann et al., 2022). Another specific issue in the study of HRI concerns the Wizard of Oz technique, in which an experimenter controls the behavior of the robot. To be able to replicate such studies, the study process and the protocol governing the wizard’s behavior need to be documented precisely (Belhassein et al., 2019).

Possibly one of the most debated methodological issues in HRI is the use of online studies that show videos of HRI to participants. We must unpack this method, since it consists of several methodical choices. First, it is important to distinguish between the recruitment of the participants and the execution of the study. Participants could be recruited online or in person. Participants can then participate in the study online from wherever they are or they could be asked to come to a specific location, such as a university lab. In both cases, computers will likely be used to play the videos and to collect survey data. Furthermore, it is also important to distinguish between interacting with a robot and viewing a video. While interacting with a robot requires in-person experiments, videos offer some distinct advantages over in-person HRI. Videos can be viewed at the convenience of the participant at home or in the laboratory.

There is still a debate dividing scholars as to whether videos can replace in-person HRI. While some studies indicate a difference in results in favor of embodied robots, such as more positive interactions and greater trust (Bainbridge et al., 2011), or in favor of video-displayed robots, other researchers believe that behaviors depend on the task (Powers et al., 2007). Li (2015) analyzed several papers and identified reporting of 39 effects in 12 studies comparing co-presence and telepresence robots. Of these 39 effects, 79% were in favor of co-presence robots (such as finding them to be perceived more positively and to elicit more positive responses), while 10% were in favor of telepresence robots. An interaction between the two groups was observed in 10% of cases. The authors report roughly similar percentages for improvements in human behavior. In their review of prosocial behavior, Oliveira et al. (2020) demonstrated this variety in responses with studies using virtual and embodied robots to trigger prosocial behaviors. With a final sample size of 19 publications presenting 23 studies, the authors indicated that 22% of these studies showed no association between physical and virtual social robots and 26% showed mixed effects. Thellman et al. (2016), in contrast, emphasize that it is not the physical presence of the robot that matters but the social presence.

### 1.1 Crowdsourcing

The combination of recruiting participants online and showing them videos online streamlines the research process. It is much quicker and cheaper than running studies with people and robots in the lab. During the COVID-19 crisis, this was practically the only way of conducting HRI studies. There are several advantages of conducting studies this way. First, conducting studies online enables researchers to recruit more participants with greater demographic diversity (Buhrmester et al., 2011). Additionally, pre-screening can be carried out to recruit participants who meet certain demographic criteria (e.g., by sharing the study only with people aged between 20 and 35). Online studies ensure that all participants get to experience exactly the same interaction, avoiding some experimenter biases and leading to consistent presentation of the stimuli (Naglieri et al., 2004). This may prevent the Hawthorne effect, i.e., the fact that humans modify some aspects of their behavior because they feel observed (Belpaeme, 2020). Finally, conducting studies online can provide more diverse participants in terms of demographics than typical American college samples, and such samples will be more representative of non-college segments of the population (Buhrmester et al., 2011). The authors of these studies also specify that, while participation rate is influenced by people’s motivations (compensation and study duration), the data obtained with this method remain at least as reliable as those collected using traditional methods.

Many scholars have examined the use of crowdsourcing services for online studies and compared them with each other or with in-person experiments. While some studies have shown that results obtained online are similar to those obtained from in-person experiments (Buhrmester et al., 2011; Bartneck et al., 2015; Gamblin et al., 2017), other studies have shown that the responses are different. Douglas et al. (2023) compared the results of recruiting participants from different online crowdsourcing sites, including Amazon’s Mechanical Turk (MTurk), Prolific, CloudResearch, Qualtrics, and SONA. They found that while Prolific and CloudResearch are the most expensive recruiting platforms, these participants were keener to pass attention checks than those recruited via MTurk, Qualtrics, and SONA, therefore providing better-quality data. High quality was attributed to the same two crowdsourcing sites; this was calculated according to various factors, including attention checks, IP address, and completion time. The authors also highlighted that recruiting participants using the SONA software took longer.

Although they identified advantages of using Prolific over MTurk, Peer et al. (2017) observed comparable data quality between both platforms. However, they highlighted the presence of naivety among Prolific and CrowdFlower participants over those recruited via MTurk, with much greater diversity and the lowest rate of dishonest behaviors also occurring on the former two platforms. The authors define the concept of naivety as a property of participants who have not become professional questionnaire-fillers who earn money this way on a daily basis. However, the use of CrowdFlower did not reproduce results that have been replicated on MTurk and Prolific. They conclude that Prolific is the best alternative to MTurk, even if the response rate is slightly lower. Adams et al. (2020) successfully replicated one of Peer et al.’s studies by comparing three sample groups: Prolific, MTurk, and a traditional student group. They obtained similar results, but support the use of Prolific over MTurk based on other factors (e.g., naivety, attention). No significant difference in terms of dishonesty was reported, contrary to Peer et al. (2017).

Gamblin et al. (2017) also compared different participant recruitment platforms, such as SONA and MTurk. They found the same patterns among SONA and in-person participants, while the results for MTurk participants varied more widely. However, people recruited via the SONA system had stronger attitudes, including racism, than the other groups, suggesting low levels of social desirability in this sample. Although these crowdsourcing platform participants seem to be a great alternative to in-person studies, the quality is not always the best, and quality controls are important.

### 1.2 Quality control

All experiments require a level of quality control. This applies to the responses received from participants as well as ensuring that the technology used, such as robots, shows consistent behavior. At times, participants might decide to randomly select answers to reduce the time they have to spend on participation. This is especially true when they participate in online studies and can chain them together to earn more money more quickly, or even try to duplicate their participation (Teitcher et al., 2015). People would therefore not answer in an optimal way. They would interpret the questions superficially and simply provide reasonable answers instead of optimizing their response, which would require cognitive effort (Krosnick, 1991). According to Krosnick (1991), satisficing increases as a function of three factors, namely, the difficulty of the task, the motivation of the respondent, and the respondent’s ability to perform the task. Hamby and Taylor (2016) examined how these factors influence the likelihood of satisficing. They found that financially motivated MTurk participants were more likely to satisfice than an undergraduate sample motivated by course credits from their university. They also reported in their first study that the three factors underlying satisficing behaviors increased the consistency and validity of responses. Thus, external motivations seem to be a reason for a drop in data quality (Mao et al., 2013).

Duplicate answers from participants can be detected by checking the participants’ IP addresses (Teitcher et al., 2015; Godinho et al., 2020; Pozzar et al., 2020). Daniel et al. (2018) proposed several strategies to improve data quality, such as improving task design and increasing the participants’ motivations, both external (e.g., incentivizing people with a bonus for good performance) and internal (e.g., comparing their performance with that of other respondents). It is important to note that incentives do not influence the response rate of online surveys (Wu et al., 2022) and that even when compensation is low, data quality does not seem to be negatively affected (Buhrmester et al., 2011).

A variety of methods are used to avoid bad actors and to elicit valid and reliable data. These range from not overburdening participants to filtering problematic responses. A common method is to include attention checks that only ask the participants to select a specific answer, such as “Select answer number three”. All participants who failed to respond to this question correctly could be excluded from further data analysis.

### 1.3 Bots

Using crowdsourcing platforms for the recruitment of participants for experiments is big business. MTurk is estimated to have at least 500,000 active users (Kuek et al., 2015). Bad actors can use automated form-fillers or bots (Buchanan, 2018; Pozzar et al., 2020; Griffin et al., 2022) to optimize their profits. In their study, Pozzar et al. (2020) analyzed low-quality data sets and respondent indicators to classify responses as suspicious or fraudulent. Out of 271 responses, none were completely of good quality. They categorized 94.5% of the responses as fraudulent and 5.5% as suspicious. More than sixteen percent could have been bots. Griffin et al. (2022) estimated that 27.4% of their 709 responses were possibly from bots. Buchanan (2018) proposed collecting data from 15% more participants to compensate for low-quality data and automated responses. This safety margin is expensive and quality control requires considerable effort.

Many web pages want to ensure that their users are humans and hence have introduced the use of a Completely Automated Public Turing test to tell Computers and Humans Apart (CAPTCHA). Users of the internet are sometimes asked while browsing a web page, for example, to click on all the pictures in a grid that include a traffic light, to prove that we are humans.

Modern online questionnaire platforms, such as Qualtrics, offer a variety of such tools to detect and prevent abuse,^1^ such as prevention of ballot box stuffing, CAPTCHAs, bot detection, and designation of some answers as spam by detection of ambiguous text or unanswered questions. However, bots might bypass these protective measures (Griffin et al., 2022; Searles et al., 2023). Metadata, such as the IP address and response time, could help prevent fraudulent respondents after the data have been collected. If a large number of responses come from the same IP address and/or the questionnaire is answered within less time than humans typically take, then the responses are likely not trustworthy. Bad actors can then use IP address disguises, such as VPNs, to avoid detection. This will continue to be a cat-and-mouse game in which bad actors will continue to come up with ways to work around detection methods and the platforms continue to introduce more sophisticated tests.

### 1.4 Large language models

One way of determining whether data come from a human being is to ask the participant to write a few sentences that justify his or her response to a previous question (Yarrish et al., 2019). This approach, and for that matter all of the abuse detection methods discussed above, are now being challenged by the arrival of large language models (LLMs), such as ChatGPT from OpenAI,^2^ BERT from Google (Devlin et al., 2019), or LLaMA from Meta (Touvron et al., 2023).

Several scholars have claimed that texts generated by these LLMs, including ChatGPT, are similar to or even indistinguishable from human-generated text (Susnjak, 2022; Lund et al., 2023; Mitrovic et al., 2023; Rahman and Watanobe, 2023). Not only can ChatGPT-4^3^ bypass a CAPTCHA by pretending to be blind,^4^ but it can also answer open-ended questions (Hämäläinen et al., 2023). The same authors showed that LLMs can be used to generate human-like synthetic data for HCI tests. In their study, they asked their participants to judge whether different texts had been generated by a human or an AI, and the participants tended to think that those generated by AIs were in fact generated by a human, with a probability of correctly recognizing AI-generated texts of 40.45%. Human texts, conversely, were correctly detected only 54.45% of the time. AI-generated responses covered similar subjects to those of human participants but with less diversity and with the presence of anomalies. While the authors propose that LLMs could be a good way of preparing exploratory or pilot studies, they warn that their abusive use in crowdfunding services could result in the data collected being unreliable.

LLMs do have a distinctive characteristic that might promote their use for abusive purposes. Creating longer texts comes at no practical increase in cost. Some platforms pay participants in proportion to their efforts: a participant who writes 1,000 words will earn more than one who only writes 10. Since LLMs can easily generate long passages of text, this is an ideal environment for abuse.

### 1.5 Readability

Stylometry could be used to detect AI-generated texts (Kumarage et al., 2023). This method corresponds to writing style analysis, including, for example, the phraseology, the punctuation, and the linguistic diversity (Gomez Adorno et al., 2018; Kumarage et al., 2023). According to these authors, phraseology corresponds to the “features which quantify how the author organizes words and phrases when creating a piece of text.” For their linguistic diversity analysis, Kumarage et al. (2023) used the Flesch Reading Ease score (Flesch, 1948; Kincaid et al., 1975). Readability is what makes some texts easier to read than others (Dubay, 2004; DuBay, 2007), and consequently this represents an estimation of the difficulty of texts (Si and Callan, 2001) and how easy it is to read them (Das and Cui, 2019).

DuBay (2007) highlights the fact that prior knowledge and reading skills might impact how easy a text is. Most readability scores refer to a ranking of the reading level a person should have in order to understand the text [see Dubay (2004), DuBay (2007) for a review on readability]. One of the most common variables used in existing formulas is the number of words, but according to Si and Callan (2001), these ignore text content. They created a model that they claim is more accurate than the Flesch Reading Ease score and more accurate in judging K-8 science web pages.

LLMs can be used to create texts about any topic on which they are prompted. For this reason, we need to know whether their required reading levels, i.e., their readability scores, are similar to those of texts written by humans. If texts generated by AI were very easy or quite difficult to read compared to their human counterparts, guessing the author of a text might be easier than if they were similar. This leads to our first research question:

**RQ1:** Is the readability of stimuli written by an AI significantly different from that of those written by a human?

While readability scores are meaningful in this context, the quality that people attribute to a text is also of great value. People might judge the quality of a text according to its required reading level, where a text that is too difficult might be judged as being of poor quality, or on the contrary, as being of very high quality. We planned to use participants’ justifications (or attention checks) provided during study participation as our stimuli. As discussed earlier, their quality is of prime importance, as they determine whether or not researchers include data from the corresponding participant. Our two next research questions were thus:

**RQ2:** What is the correlation between readability and user-perceived quality of the stimuli?
**RQ3:** Do participants rate the quality of text written by an AI differently from that of text written by a human?

### 1.6 The imitation game

Testing whether humans can tell the difference between a human and a machine is often referred to as a Turing Test. Alan Turing proposed the Imitation Game method in 1950 to test whether machines can think (Turing, 1950). In this test, a machine (Player A) and a human (Player B) are behind a door. An interrogator (Player C) communicates with them by slipping a piece of paper under the door. The goal of the machine is to imitate the human. The human has the goal of helping the interrogator. The conversations the players may have are unconstrained and can take as long as the interrogator wants. This test is repeated with many interrogators, and if they are unable to reliably tell the human player apart from the machine, then Turing argues that the machine is able to think. Turing proposed several variations on the test (Turing, 2004; Turing et al., 2004), which go beyond the scope of this paper. The interested reader might consult Copeland and Proudfoot (2009) for an extended discussion.

The Turing Test was designed to test a machine’s ability to think. However, as time has passed, the terms “Turing Test” and “imitation game” have often been used interchangeably in various research contexts. Such imitation games are commonly referred to as “Turing-style tests.” One of the best-known Turing-style tests is the CAPTCHA (Ahn et al., 2003). This test is often referred to as a “reverse Turing Test,” since the role of the interrogator is carried out by a machine rather than a human. The Loebner Prize Competition used another variation on Turing’s test to identify the best chatbot (Moor, 2001). The Feigenbaum Test (Feigenbaum, 2003) represents yet another Turing-style test. Feigenbaum suggests employing the Imitation Game mechanism to assess whether a machine possesses professional-level quality. The judge’s challenge in the Feigenbaum Test is to determine whether the machine shows the appropriate level of expertise in a specific domain. The judge must of course be an expert in the field. The quality of text is still an important criterion for distinguishing between humans and machines.

Initial results indicated that GPT-3 could pass a variation on Turing’s test (Argyle et al., 2023). The participants in their study correctly recognized 61.7% of a human-generated list and 61.2% of a GPT-3-generated list. Scholars do not always obtain similar results. Gao et al. (2023) used not only human participants but also the GPT-2 Output Detector to identify the authors of human- and AI-generated scientific abstracts. While participants correctly identified 68% of AI-generated abstracts and misidentified 14% of human-written abstracts as AI-generated, the automatic AI detector had an accuracy of 99% in identifying AI-generated abstracts as AI. However, not only have all studies used stimuli of different origins, but LLM skills also progress quickly, and recent results might already be obsolete. Here, we are interested in the ways in which people can detect the authorship of texts that people present as justifications for their answers and as data quality checks. This leads to our next research questions:

**RQ4:** How accurately can participants identify the authors of the stimuli?
**RQ5:** How accurate and reliable are automatic AI detection systems?
**RQ6:** What is the relationship between the accuracy of AI detection systems and the accuracy of human participants?

Interestingly, scholars have attempted to discern the criteria that participants use to make their decisions (Guo et al., 2023; Hämäläinen et al., 2023). However, to our knowledge, no study so far has combined LLMs with AI obfuscation systems, which are supposed to make AI-generated texts undetectable, in the context of scientific experiments. Thus, our final two research questions were:

**RQ7:** What criteria are participants using to distinguish text generated by an AI from that written by a human?
**RQ8:** Is Undetectable.AI able to overcome AI detection systems?

### 1.7 Research objectives

Participants have many legitimate reasons for participating in online studies, which include earning money, satisfying curiosity, and even for entertainment. However, it only takes a handful of bad actors with some programming skills to compromise the whole system. With the rise of LLMs and other forms of artificial intelligence (AI), we need to know how good our fraud detection systems are. The considerable progress in AI and LLMs in recent months, and in systems aiming to make their detection more difficult, highlights the potential threat posed. Their actions are no longer limited to the generation of responses in questionnaires; their capabilities extend to the automation of many tasks, such as through Auto-GPT.^5^ It is of utmost importance that we can continue to control the quality of responses in online studies. This includes the use of automatic detection systems and people’s ability to identify the author of a text.

## 2 Methods

We conducted a within-participants study in which the author of textual stimuli was either an AI or a human.

### 2.1 Participants

Forty-two students from the University of Canterbury were recruited for this study. Twenty-one of them were male and 20 were female, while one person declared their gender to be different. Their ages ranged from 18 to 31, with an average of 20.9 years. The study was advertised on a news forum of the Department of Computer Science and Software Engineering; hence, many students reported that they studied this subject.

### 2.2 Setup

The study was conducted in a laboratory space at the University of Canterbury. The room contained six computer workspaces that were separated by partitions. The participants were not able to see or interact with each other while answering the questionnaire. Each workspace consisted of a 24-inch monitor with a screen resolution of 1920 × 1080 pixels. Participants were seated approximately 60 cm away from the screen. The experiment was conducted using the web-based questionnaire service Qualtrics.^6^ The web browser was set to kiosk mode, which prevented participants from conducting any operation other than answering the questionnaire.

### 2.3 Stimuli

This experiment required text written by either humans or AI. This text had to come from an actual previous scientific experiment in order to align with the context of our study. A list of the stimuli is provided in Supplementary Appendix SB.

#### 2.3.1 Selection of human text

We used text from a previous HRI experiment which used a 2 × 2 between-participants setup. This study is currently available as a preprint (Vonasch et al., 2022); its exact focus is irrelevant to the study at hand. The only requirement was that the text was written by participants in a scientific study for the purpose of quality control. We refer to the study from which we collected our text as the source study.

In the source study, participants had to respond to an imaginary interaction. For example, the following vignette could be presented to participants:

You are thinking about buying a used car from a dealership downtown. The sales representative is a robot named Salesbot that has learned from experience that customers are more likely to buy when it cuts the price. Salesbot shows you a car you think you might like. “We have it listed at $5000, but for you I can make a special deal on it. How about $700?”

Four types of interaction were used in this source study with two independent variables: agent (human seller or robot seller) and discount (low or high). Participants then had to rate on a Likert scale whether they would buy the car. Next, they were asked to justify their decision. An example response was:

From what this bot is telling met [*sic*], I can gather two things: I’m either being swindled or I [*sic*] this is borderline theft. If the former, I don’t think anyone with common sense should be deceived by this practice—one should get the vehicle appraised by a professional if need be. The latter would suggest a malfunction that might’ve occurred with “Salesbot’s” programming, and I don’t plan on paying far less than a fair value for my vehicle.

The example above shows that the participant clearly considered the interaction. If a participant had written nonsense or just a single word, their data would normally be considered unreliable and hence excluded from further analysis. Again, the exact details of the source study are only of tangential relevance to the study at hand.

**FIGURE 1:**
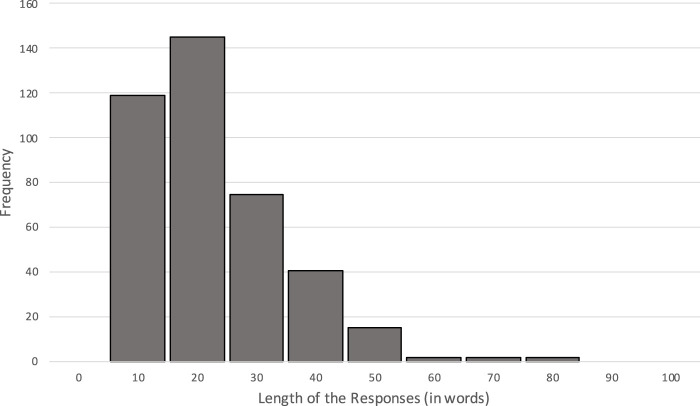
Distribution of response lengths in the source experiment.

The source study used for sampling of our human stimuli collected 401 responses across its four conditions. We focused on the justifications written by the participants for the purpose of quality control. The lengths of the 401 justifications did not follow a normal distribution (see Figure 1).

In order to select our ten human stimuli, it was necessary to determine whether a specific condition led to more elaborate justifications by the participants, since controlling the length to be similar to that of the AI stimuli would improve validity. To do so, we conducted an ANOVA to test whether the lengths of the justifications written by the participants differed across the four conditions of the source experiment. The results indicated an effect of discount (*p* = 0.003) but no effect of agent (*p* = 0.102). Pairwise comparisons indicated that the discount effect was in favor of the low discount, with more words written in this condition (*M* = 20.18) compared to the high-discount condition (*M* = 16.88), *p* = 0.003. However, the mean difference was very small (less than 3.5 words), and thus a small effect size *η* = 0.022 was reported. We therefore decided to select our human stimuli based on length, i.e., to use the ten longest participant justifications. The selected texts ranged from 47 to 76 words in length, with an average of 57.7 words (*SD* = 10.99, median = 55.50). This allowed us to generate the AI stimuli based on the same criteria (condition and length).

#### 2.3.2 Generation of AI text

We used ChatGPT^7^ and Undetectable.AI^8^ to generate the AI text. The latter is necessary for avoiding discovery by automatic AI detection systems that experimenters could use to filter out AI-generated responses. It would have been easy to ask AI tools to simply rephrase the human stimuli. However, this would not align with the context of our study. If bad actors wanted to generate automatic responses for online experiments, they would not have access to responses from human participants. Instead, they would need to use the context of the online study to generate responses. Hence, we only used the information available to the participants in the source study to generate justification texts. Moreover, we provided ChatGPT with exactly the same context in which each of the ten selected human stimuli was collected. The human and AI stimuli can therefore be related to each other.

We developed a strict protocol for generating text using AI tools. The full protocol is available in Supplementary Appendix SA. In short, we used the following steps:

1. For each of the ten selected human stimuli texts, we recorded the context in which they were collected.
2. We prompted ChatGPT to play the role of a participant in an experiment.
3. We prompted ChatGPT with the context of the source study, including the text vignette and the Likert scale.
4. We asked ChatGPT to generate justifications for all of the possible Likert ratings, of the length of the corresponding human text.
5. We recorded the justification text that corresponded to the human response.
6. We used Undetectable.AI to rephrase the AI text using the “University” readability setting.

Each of the ten human stimulus texts was therefore matched to an AI-generated stimulus text. The corresponding AI text for the example presented above (see Section 2.3.1) was:

My position regarding Salesbot offering a different pricing strategy differs from their original listing because it may compromise transparency during negotiations between both parties. A reliable purchase involves honesty and fairness on both sides. Especially when it comes to buying used cars. By intentionally inflating prices this puts customer trust at risk while also causing confusion about what exactly they will receive from their investment in this vehicle thereby leading possible frustration on their part. Therefore. [*sic*] My suggestion is that Salesbot should instead focus on being upfront about pricing by adhering strictly to their original listing value.

The responses received from ChatGPT were approximately the same length as the corresponding human stimulus texts. To ensure that the AI-generated text could not be detected by machines, we asked Undetectable.AI to rephrase the text. This slightly changed the length of the text. While the average length of the ChatGPT-generated stimuli was 58.6 words (*SD* = 13.68, median = 54.50), that of the final stimuli generated by Undetectable.AI was 71.2 words (*SD* = 23.14, median = 64.00). Although we expected to obtain stimuli of the same length, to our knowledge, there is no option to constrain the length of the output of Undetectable.AI. A Kruskal–Wallis test was conducted to examine the differences in text length between the three author groups (Human, ChatGPT, and Undetectable.AI). No significant difference was found between these three groups, *H*(2) = 2.60, *p* = 0.273. Equivalence tests were conducted for each pair: Human–ChatGPT [*t*(17.2) = −0.0721, *p* = 0.528], Human–UND [*t*(12.9) = −1.60, *p* = 0.934], and ChatGPT–UND [*t*(14.6) = −1.42, *p* = 0.912]; the equivalence hypothesis was not rejected.

The participants in our study were located in New Zealand and hence would predominantly be familiar with British English spelling. The companies operating ChatGPT and Undetectable.AI are America-based, and hence it is conceivable that their software might promote American English spelling. We manually checked that the AI stimuli were neutral in terms of linguistic variation, avoiding any explicitly American or British dialect usage (e.g., spelling variations such as “s” vs. “z” in, for example, the word “humanize”). This was done to ensure that participants, whatever their dialect, could understand the sentences without cultural or ethnic bias, and to reduce the risk of their judging sentence quality based on these variations.

### 2.4 Procedure

The study lasted approximately 20 min and participants were compensated with a $10 gift voucher.

#### 2.4.1 Welcome and consent

After welcoming the participants, the experimenter provided a description of the study and a consent form to the participants. The description informed the participants in broad terms that the study aimed to determine how people evaluate sentences in the context of human–robot interaction. After agreeing to take part in the study, the participants were seated in front of a computer.

#### 2.4.2 Phase 1: quality

**FIGURE 2:**
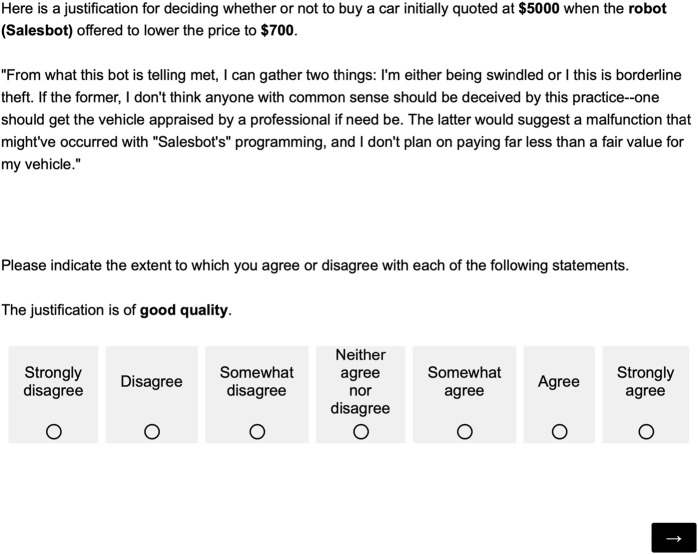
Questionnaire used to measure the quality of the text.

In the first phase, the participants were asked to rate the quality of all the text stimuli. Prior to rating the 20 stimuli, a training session with two training trials using two contexts and associated texts was shown to the participants. At the end of the training session, the participants were informed that they could now ask the experimenter any questions that they might have. Subsequently, the participants were shown the 20 stimuli text, one at a time and in a randomized order. The authorship of each text, either human or AI, was *not* revealed to them. An example of such a task is shown in Figure 2. The stimulus texts were accompanied by the context in which they were generated. The generated text was referred to as a “justification” for the contextual information provided. After completing the first phase, the participants were then thanked and invited to start the second phase.

#### 2.4.3 Phase 2: imitation game

**FIGURE 3:**
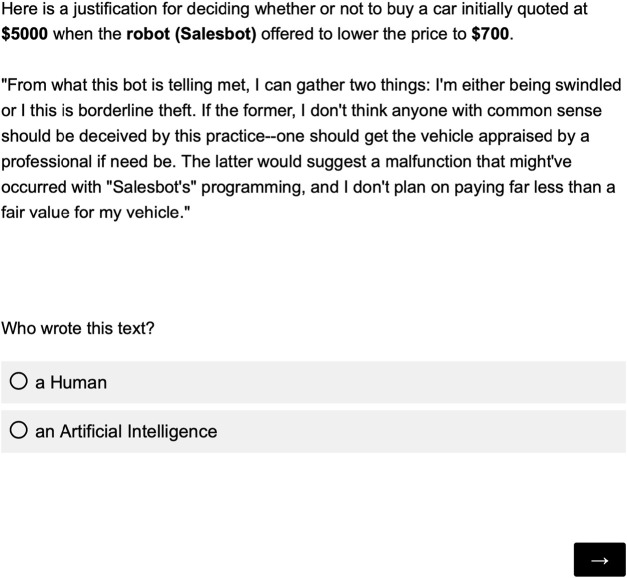
Questionnaire used to obtain judgements on the authorship of the text.

In the second phase of the study, participants were informed that each of the twenty stimulus texts were generated either by a human or an AI. They reviewed each text and its associated contextual information one by one in a randomized order. They were asked to identify the authorship of the stimulus text (see Figure 3).

Prior to responding to the 20 stimuli, participants went through two training trials. After the training, the participants could ask the experimenter any questions they might have. After making their choices for all twenty stimuli, participants were asked in an open-ended question to explain the criteria they used to decide whether each text was written by a human or an AI. They were then thanked for completing the second phase and invited to complete the third phase.

#### 2.4.4 Phase 3: demographics and debriefing

In the third and final phase of the experiment, demographic data, such as gender, age, and field of study, were collected. The participants were then debriefed. They were informed that the authorship of the stimulus texts had been unavailable to them in the first phase. This might be considered an omission of truth and therefore a mild form of deception. The necessity of this approach was communicated to the participants in accordance with the ethical standards of the university. Once they had been informed, they had the option of withdrawing their data without having to justify their decision. None of the participants did, which leads us to believe that the omission of truth in the first phase was acceptable to them in the context of the purpose of this study.

### 2.5 Measurements

#### 2.5.1 Data quality

We recorded the completion times of the participants in our study to check for problematic responses.

#### 2.5.2 Text quality

The quality of all stimulus texts was measured using both readability scores and a Likert scale rating.

We used the Arte software^9^ to calculate the following readability scores for each of the 20 stimuli:

• Flesch Reading Ease (Flesch, 1948; Kincaid et al., 1975)
• Simple Measure of Gobbledygook or SMOG (Mc Laughlin, 1969)
• Dale–Chall (Chall and Dale, 1995)
• Automated Reading Index, or ARI (Smith and Senter, 1967; Kincaid et al., 1975).

We also chose to use the Gunning fog index (Gunning, 1952), but this was not available in the Arte software. To do so, we used another site^10^ created exclusively for this index.

Grammarly^11^ was used to count the number of spelling and grammar mistakes in the stimuli. Before use, Grammarly first asks what the objectives are in terms of audience, formality, and intent. To better align Grammarly to the other readability scores used in this study, the target audience was set in Grammarly’s default settings. The default audience is “knowledgeable” and the tool focuses on reading and comprehension.

The quality of the stimuli was measured by asking participants to respond to the statement “The justification is of good quality” on a seven-point Likert scale, ranging from “strongly disagree” to “strongly agree” (see Figure 2).

#### 2.5.3 Imitation game

We used both automatic AI detection systems and the ratings of participants to identify the authorship of the stimuli. The stimuli were analyzed by the following AI detection systems (use of the software between 7 and 27 June 2023; collection of descriptive information on 11 July 2023):

1. Undetectable.ai.^12^ This software is not only an AI detector but also a text humanizer. It is possible to choose to modify texts to make them more readable, more human, or a mix of both.
2. GPTZero.^13^ This software aims to identify the author of a text using average perplexity and Burstiness scores (a measurement of the variation in perplexity).
3. Copyleaks.^14^ This software is described as being able to sniff out the signals created by AI. The site claims: “The AI Content Detector can detect content created by most AI text generators and text bots including the GPT4 model, ChatGPT, Bloom, Jaspr, Rytr, GPT4 and more.”
4. Sapling.^15^ This software is said to provide the “probability it thinks each word or token in the input text is AI-generated or not.”
5. Contentatscale.^16^ This software uses watermarking to find out whether a text was written by an AI or a human.
6. ZeroGPT.^17^ This software is said to use “DeepAnalyse Technology.”
7. HugginFace.^18^ This software analyzes the perplexity/unpredictability of the text and identifies human-like patterns using chunk-wise classification.

The AI detectors claim to have good detection accuracy, but most of the time do not explain precisely how their success has been measured or how they were trained. There are more AI detection systems available, but some, such as Originality,^19^ did not offer a free trial. Others require a minimum number of words or characters that was above the length of our stimuli. The OpenAI Classifier,^20^ for example, was therefore excluded from our list. In addition, we also tested the original ChatGPT stimulus texts to test whether the use of Undetectable. AI was necessary.

The participants in our study were asked to identify the authorship of all stimulus texts during the second phase of the experiment (see Figure 3).

## 3 Results

### 3.1 Descriptive

#### 3.1.1 Participants

The participants’ fields of study were distributed as follows: 30 in computer science, 3 in engineering, 3 in science, and 6 in other fields of study.

#### 3.1.2 Data quality

All data collected met our quality criteria. All of the participants completed the study in a reasonable amount of time. They showed coherence in their responses to the open-ended questions, and showed no signs of “straightlining.” Qualtrics did not flag any of the responses as suspicious. Surprisingly, one participant enrolled twice and claimed not to have participated before. As his data were of good quality for the first participation, they were retained for analysis, but he was prevented from participating a second time. It is not impossible to think that his motivation for taking part a second time was related to the compensation.

#### 3.1.3 Stimulus grammar quality

Unlike typical human-generated texts, text generated by AI, such as through ChatGPT, does not normally contain spelling or grammar mistakes. We used the Undetectable.AI software to obscure the AI authorship, but we had no information on how exactly this was achieved due to the limited documentation available. Adding spelling and grammar mistakes would have been an option. We therefore used the Grammarly software^11^ to count the number and type of mistakes (see Table 1). Since the AI stimuli were generated using ChatGPT and transformed with Undetectable.AI, it was interesting to determine whether there was a difference between the two tested groups of texts and the intermediate ChatGPT texts in terms of spelling and grammar errors. Thus, TOST paired-sample t-tests were conducted to see whether there was any difference in the number of spelling and grammar mistakes between the three groups of stimuli (human, Undetectable.AI, and the intermediate ChatGPT texts). Bounds of −0.5 and 0.5 were used. No significant difference was found between the human and Undetectable.AI stimuli [*t*(9) = 0.446, *p* = 0.333, *d* = 0.212]. However, Grammarly reported no spelling or grammatical mistakes for the ChatGPT stimuli. Although paired-sample t-tests indicated a significant difference in terms of the quantity of spelling and grammar mistakes, not only between the intermediate ChatGPT stimuli and the human stimuli [*t*(9) = 2.74, *p* = 0.023, *d* = 0.791], but also between the intermediate ChatGPT stimuli and the Undetectable.AI stimuli [*t*(9) = 3.88, *p* = 0.004, *d* = 1.121], TOST analyses did not result in rejection of the equivalence hypotheses; *t*(9) = 1.37, *p* = 0.898, and *t*(9) = 2.39, *p* = 0.980, respectively.

**TABLE 1:** Count of spelling and grammar mistakes between the three groups of stimuli, as detected by Grammarly.

Stimuli	Human	Undetectable.AI	ChatGPT
1	2	3	0
2	2	0	0
3	0	1	0
4	3	1	0
5	0	2	0
6	2	2	0
7	0	0	0
8	1	2	0
9	0	2	0
10	0	0	0
Total	10	13	0

### 3.2 Readability and quality

#### 3.2.1 Question 1: is the readability of stimuli written by an AI significantly different from that of those written by a human?

The average readability scores are presented in Table 2. A paired-sample t-test was conducted in which author type (human or AI) was the within-participants factor for all ten sentences. To do so, Bonferonni correction was applied and the initial alpha value of 0.05 was reduced to 0.01. The paired-sample tests revealed a significant difference in readability for the Flesch Reading Ease score, *t*(9) = 8.87, *p* < 0.001, *d* = 2.805, with higher scores for the human-author texts (*M* = 80.132, *SD* = 10.364) compared to the AI-author texts (*M* = 39.172, *SD* = 13.528), 95% CI [30.514, 51.406]. This score means that the human-generated texts, on average, were easy for an 11-year-old child to read, unlike those generated by AI, which were more suitable for someone with a college reading level.

**TABLE 2:** Average readability scores for human and AI stimulus texts.

Readability scale	Human	AI
Flesch	80.132	39.172
SMOG	8.100	15.000
Dale–Chall	6.539	10.122
Fog	8.868	15.403
ARI	6.141	13.541

Similar differences between the human and AI texts were found for the four other readability scores, namely, the SMOG [*t*(9) = −5.86, *p* < 0.001, *d* = 1.852], Dale–Chall [*t*(9) = −5.66, *p* < 0.001, *d* = 1.789], Fog [*t*(9) = −4.57, *p* = 0.001, *d* = 1.446], and ARI [*t*(9) = −3.89, *p* = 0.004, *d* = 1.230] indices. On average, the readability scores showed that the texts written by humans were comprehensible to children aged 13–14 years (10–11 years in the case of the ARI), whereas a college reading level (17–18 years in the case of the ARI) was required for texts written by an AI. In other words, according to these indices, the texts written by humans tended to be easier to understand and were written at a lower educational level than those generated by artificial intelligence.

#### 3.2.2 Question 2: what is the correlation between readability and user-perceived quality of the stimuli?

We performed a regression analysis of the average user-perceived quality of the 20 stimuli and their readability scores (data are presented in Table 3). While the analyses highlighted weak correlations between user-perceived quality and each readability score, none of these were significant (*p* > 0.05).

**TABLE 3:** Readability scores and average user-perceived quality per stimulus and author.

Stimulus	Author	Flesch	SMOG	Dale–Chall	Fog	ARI	Quality
1	Human	63.22	11	9.42	10.76	12.66	4.50
2	Human	90.78	7	7.81	4.71	1.15	3.38
3	Human	86.22	8	6.63	8.96	5.48	4.64
4	Human	86.11	7	6.42	8.84	5.15	5.33
5	Human	71.03	9	7.09	6.75	1.83	5.71
6	Human	88.06	7	1.88	10.20	7.20	5.31
7	Human	64.39	11	6.47	12.10	11.36	5.52
8	Human	80.25	7	6.14	10.43	8.62	5.52
9	Human	81.13	8	7.77	8.82	5.83	5.88
10	Human	90.13	6	5.76	7.12	2.13	4.40
1	AI	28.20	15	9.81	15.54	14.18	4.52
2	AI	27.79	17	10.43	20.27	17.48	5.55
3	AI	42.11	14	10.05	16.27	16.76	4.67
4	AI	50.02	14	9.82	15.21	13.34	6.43
5	AI	18.11	16	12.26	18.39	16.46	4.33
6	AI	60.99	11	9.57	12.00	7.98	5.19
7	AI	43.50	13	10.47	16.07	14.21	5.67
8	AI	54.51	22	9.36	10.58	8.57	5.50
9	AI	36.91	13	9.24	14.59	12.16	5.79
10	AI	29.58	15	10.21	15.11	14.27	6.02

Notes. The column “Quality” corresponds to the average rating of user-perceived quality.

#### 3.2.3 Question 3: do participants rate the quality of text written by an AI differently from that of text written by a human?

**FIGURE 4:**
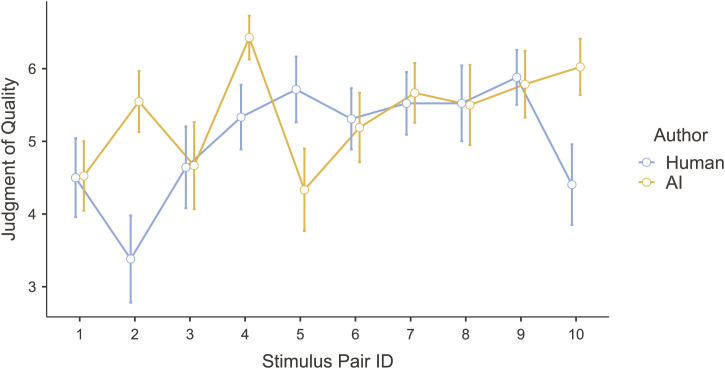
User-perceived quality of both AI-generated and human-generated texts.

A repeated-measures ANOVA was conducted to test what predicts how people perceive the quality of a text: does the type of author matter (i.e., human vs. AI), and is this effect the same or different for each of the stimulus pairs we examined? Although the data were distributed non-normally, violating an assumption of the ANOVA, prior work demonstrates that violations of this assumption almost never influence the outcome of an analysis (Blanca et al., 2017). Three factors (author type, stimulus pair, and their interaction) were used to predict text quality. There were significant effects of author, stimulus pair, and their interaction (see Table 4), meaning that the user-perceived quality of text depended on whether the author was a human or an AI, but this differed for each stimulus pair (see Figure 4). For six of the stimuli, user-perceived quality did not differ between human and AI authors. For three of the stimuli, the AI-generated texts were perceived to be of higher quality, and for one of the stimuli, the human text was perceived to be of higher quality. Qualitative analysis of these texts explained why: in the cases in which the AI text was judged to be of higher quality, the human text was written informally, with noticeable typos and incomplete sentences. In the case in which the human sentence was judged to be of higher quality, the AI sentence included copious meaningless jargon (Rudolph et al., 2023).

**TABLE 4:** Detailed results of repeated-measures ANOVA.

	Sum of squares	df	Mean squares	F	p
Stimulus	207.0	9	22.99	10.22	$<0.001^{*}$
Residual	830.4	369	2.25		
Author	25.0	1	25.03	5.56	0.023*
Residual	184.7	41	4.51		
Stimulus × Author	194.8	9	21.64	10.68	$<0.001^{*}$
Residual	748.0	369	2.03		

*: *p* < 0.05. Note. There were 10 pairs of stimuli, in which one was written by a human and one by an AI.

### 3.3 Imitation game

#### 3.3.1 Question 4: how accurately can participants identify the authors of the stimuli?

The 42 participants were asked to identify the authorship for each of the 20 stimuli, adding up to 840 data points. The confusion matrix in Table 5 summarises the results of this Imitation Game. The True Positive (TP) cell, corresponding to the number of successful attempts in which an AI author was correctly identified, is equal to 307. The number of successful attempts in which a human author was correctly identified corresponds to the True Negative (TN) cell and is equal to 333. Two cells correspond to the errors that participants made. On the one hand, the False Positive (FP) cell, corresponding to Type I errors (i.e., the identification of human-generated stimuli as generated by an AI), is equal to 113. On the other hand, the False Negative (FN) cell, corresponding to Type II errors (i.e., the identification of AI-generated stimuli as written by a human), is equal to 87. Dividing the number in each table cell by the total number of attempts provides the percentages associated with the cell number.

**TABLE 5:** Confusion matrix of the participants’ identifications for all 840 data points.

		Identification		
		Human	AI	Total
Author	Human	TN 333 (79.3%)	FP 87 (20.7%)	420 (100.0%)
	AI	FN 113 (26.9%)	TP 307 (73.1%)	420 (100.0%)
	Total	446 (53.1%)	394 (46.9%)	840 (100.0%)

Note*.* Percentages correspond to the value as a proportion of the total indicated in the cell on the corresponding row.

Based on these values, we can calculate further indicators, such as accuracy, precision, recall, specificity, and F1 score. The accuracy, i.e., the proportion of correctly identified stimuli out of all stimuli in the experiment, was equal to 76.19%, 95% CI [73.16, 79.03]. Precision, the number of correct identifications of AI out of all stimuli identified as AI, was 77.92%, 95% CI [73.49, 81.92].

The recall value represents the percentage of correct identifications of the AI-generated stimuli. In this case, participants correctly identified 73.10% of the AI-generated stimuli, 95% CI [68.58, 77.28]. Finally, the participants correctly identified 79.29% of the human-generated stimuli, 95% CI [75.09, 83.06]; this is known as specificity. The participants were 6.19% better at detecting human-generated stimuli than at detecting AI-generated stimuli.

Precision, recall, and specificity only consider either FN or FP, but never both at the same time. Hence, we were missing an indicator that considers the balance of FN and FP. The F1 score fills this gap by calculating the precision–recall harmonic mean, which depends on the average of Type I (FP) and Type II (FN) errors. The F1 score was 75.43%, 95% CI [70.95, 79.53].

Performing a *χ* ^2^ test allowed us to examine the association between the author of a stimulus and the participants’ identifications of its authorship. This non-parametric test is very robust. The *χ* ^2^ test showed a significant association between these variables [*χ* ^2^(1) = 231.36, *p* < 0.001]. The effect size, corresponding to Cramer’s *V* = 0.525, indicated a strong association between the author and the participants’ identifications.

**FIGURE 5:**
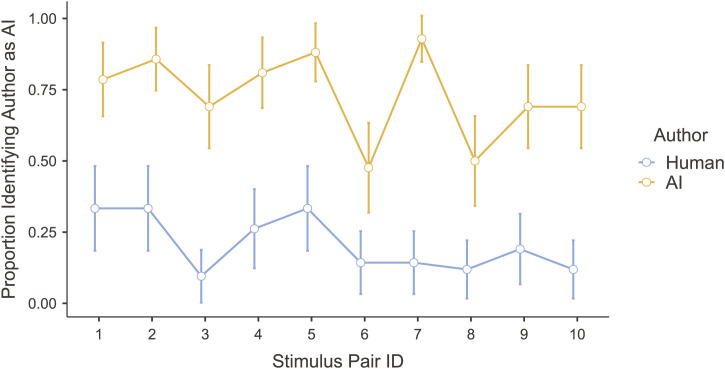
Proportion of participants judging that each sentence was written by AI (as opposed to by a human). Each pair of dots represents the pair of sentences created with the same prompt.

Because the texts were created in conceptually related pairs, we conducted a repeated-measures ANOVA with stimulus pair ID, author type, and their interaction as predictors of judgements that the author was human or AI. Because of a violation of the sphericity assumption, we conducted a Heynh–Feldt corrected analysis. The strongest predictor of these judgements was author type [*F*(1, 41) = 137.50, *p* < 0.001], although the effects of both stimulus pair ID [*F*(7.66, 313.94) = 6.72, *p* < 0.001] and the interaction [*F*(8.64, 354.14) = 2.13, *p* = 0.028] were significant, indicating that people’s judgements of human versus AI authorship depended on the particular texts they were judging. As shown in Figure 5, for every stimulus pair, people were more likely to judge the AI-written text as written by AI and the human text as written by a human, with an overall effect size of *η* ^2^ = 0.275. Thus, 27.5% of the variance was explained by author type. A further 4.6% was explained by stimulus pair ID, and 1.4% by the interaction of stimulus pair and author type. Thus, people’s judgements of the texts’ authorship largely depended on the actual author, but some texts were easier to categorize than others.

We conducted a mixed-effects logistic regression analysis to better understand the relationship between the authorship of the stimuli and the participants’ identifications of their authorship. The model included a fixed effect of the variable of author (either human or AI) and two random effects: participant (labeled PersonID) and stimulus pair (among the ten pairs of sentences as described in Section 2.3, labeled StimulusPairID). The author variable was coded as 0, representing a human, or 1, representing AI. The inclusion of the participant factor enabled us to take interpersonal differences into account, and the inclusion of the stimulus pair factor allowed us to take differences between the pairs of texts into account.

Equation 1 shows our mixed-effects linear logistic regression model, which we will refer to as CA (Correctness–Author). *Y* represents the correctness of identification, which is a binary variable that indicates whether the participants correctly identified the authorship of the stimuli. We coded a correct identification as 1 and an incorrect identification as 0. This differs from the raw identification score in Table 5 in that it does not encode the raw response (human or AI) but whether this choice was correct. $\text{Pr}(\text{Y}=\text{AI}\left.|y\right)$ is the probability of occurrence of a correct identification. Furthermore, the model included ${u_{0}}_{\text{PersonID}}$ and ${u_{0}}_{\text{StimulusPairID}}$ as random effects. It also included Author (either human or AI) as the independent factor.

$$\begin{array}{ll} & \text{Pr}\left(Y=\text{Correct}|y\right)=\frac{1}{1+e^{-y}}\\ & y=\text{Logit}\left(\frac{\text{Pr}\left(Y=\text{Correct}|\text{Author},{u_{0}}_{\text{PersonID}},{u_{0}}_{\text{StimuliPairID}}\right)}{1-\text{Pr}\left(Y=\text{Correct}|\text{Author},{u_{0}}_{\text{PersonID}},{u_{0}}_{\text{StimuliPairID}}\right)}\right)\\ & \;=\beta_{0}+\beta_{1}\times \text{Author }+{u_{0}}_{\text{PersonID}}+{u_{0}}_{\text{StimuliPairID}} \end{array}$$ (1)

Table 6 presents the variance and standard deviation of the random effects on the intercept of the CA model.

**TABLE 6:** Variance, standard deviation, and confidence interval of the random effects for the CA model.

Groups	Name	Variance	Std. Dev.	Std. Dev. 95% CI
PersonID	(Intercept)	0.391	0.625	[0.396, 0.913]
StimulusPairID	(Intercept)	0.060	0.245	[0.000, 0.561]

Table 7 shows the estimates for the fixed effect and intercept for the CA model. Both effects were statistically significant with *p* < 0.05.

**TABLE 7:** The estimated value, standard error, odds ratio, confidence interval, z-value, and *p*-value of the fixed effect for the CA model.

Effect	Estimate	Std. Error	Odds ratio	95% CI	z value	*p* value
(intercept)	1.465	0.179	4.328	[1.118, 1.845]	8.199	< 0.001*
Author	−0.370	0.168	0.690	[−0.706, −0.039]	−2.202	0.028*

Note: * indicates a *p*-value below 0.05.

The results in Table 7 indicated a main effect of author on correctness. The probability of participants correctly identifying AI-generated text (74.92%) was below that of their correctly identifying human-generated text (81.23%). Moreover, examining the odds ratios (ORs) provides further insight into how the fixed effect affects the participants’ correctness. The OR for Author (0.69) indicated that the odds of correctly identifying the authorship of the stimuli decreased by 31% (95% CI [0.497, 0.960]) for AI-generated stimuli compared to human-generated stimuli.

Equation 2 shows how recall (74.92%, 95% CI [67.47, 81.45]) and specificity (81.23%, 95% CI [74.50, 80.83]) can be estimated using the logistic regression model. These estimates were slightly above those calculated based on the confusion matrix in Table 5, which were 73.10% and 79.29%, respectively. Moreover, we also calculated values for accuracy (78.08%, 95% CI [70.99%, 81.64%]), precision (79.97%, 95% CI [72.57%, 80.95%]), and F1 (77.36%, 95% CI [69.93%, 81.20%]).

Notice that the specificity and recall values based on the confusion matrix in Table 5 were calculated slightly differently from their calculation in the logistic regression model. Recall represents *Pr*(*Y* = *Correct*|*Author* = *AI*), which means the probability of correct identification of texts written by the AI. In the mixed-effects logistic regression, recall was calculated while taking the participant and stimulus pair into account $\left(Pr\left(Y=Correct|Author=AI,{u_{0}}_{\text{PersonID}}=0,{u_{0}}_{\text{StimuliPairID}}=0\right)\right)$ (see Eq. 2). This represents the probability of correct identification of texts written by the AI taking into account interpersonal differences and differences between pairs of stimuli. Therefore, the results for these statistics calculated as part of the logistic regression model are overall slightly above those calculated based on the confusion matrix in Table 5.

$$\begin{array}{ll} & \text{Specificity}=\text{Pr}\left(Y=\text{Correct}|\text{Author}=\text{Human}=\text{0},{u_{0}}_{\text{PersonID}}=\mu_{\text{PersonID}}=0,\right.\\ & \;\left.{u_{0}}_{\text{StimulusPairID}}=\mu_{\text{StimulusPairID}}=0\right)=\frac{1}{1+e^{-\left(1.4651-0.3704\times 0\right)}}\\ & \;\;\;\;\;\;\;\;\;=0.8123=81.23\%\\ & \text{Recall}=\text{Pr}\left(Y=\text{Correct}|\text{Author}=\text{AI}=1,{u_{0}}_{\text{PersonID}}=\mu_{\text{PersonID}}=0,\right.\\ & \;\left.{u_{0}}_{\text{StimulusPairID}}=\mu_{\text{StimulusPairID}}=0\right)=\frac{1}{1+e^{{-\left(1.4651-0.3704\times 1\right.}^{)}}}\\ & \;\;\;\;\;\;\;\;\;=0.7492=74.92\% \end{array}$$ (2)

We included several other measurements in our experiment and hence conducted a second mixed-model logistic regression analysis to explore their relationships. We decided to include the length of the stimulus text as a fixed effect, since it was not possible to completely control this factor. The process we used to generate the stimuli is described in Section 2.3. Second, we included the user-perceived quality of the texts (labeled QualityScore). The measurement of this variable is described in Section 2.5.2. The saturated exploration model, which we will refer to as CALQ (Correctness–Author–Length–Quality), is specified in Eq. 3.

$$\begin{array}{ll} & \text{Pr}\left(Y=\text{Correct}|y\right)=\frac{1}{1+e^{-y}}\\ & y=\text{Logit}\left(\frac{\text{Pr}\left(Y=\text{Correct}|\text{Author},\text{Length},\text{QualityScore},{u_{0}}_{\text{PersonID}},{u_{0}}_{\text{StimulusPairID}}\right)}{1-\text{Pr}\left(Y=\text{Correct}|\text{Author},\text{Length},\text{QualityScore},{u_{0}}_{\text{PersonID}},{u_{0}}_{\text{StimulusPairID}}\right)}\right)\\ & \;=\beta_{0}+\beta_{1}\times \text{Author}+\beta_{2}\times \text{Length}+\beta_{3}\times \text{Author}\times \text{Length}+\beta_{4}\times \text{QualityScore}\;+\\ & \;+\beta_{5}\times \text{Author}\times \text{QualityScore}+\beta_{6}\times \text{Length}\times \text{QualityScore}+{u_{0}}_{\text{PersonID}}+{u_{0}}_{\text{StimulusPairID}} \end{array}$$ (3)

Table 8 presents the variance and standard deviation of the random effects on the intercept for the CALQ model.

**TABLE 8:** Variance, standard deviation, and confidence interval of the random effects for the CALQ model.

Groups	Name	Variance	Std. Dev.	Std. Dev. 95% CI
PersonID	(Intercept)	0.429	0.655	[0.420, 0.952]
StimuliPairID	(Intercept)	0.166	0.407	[0.058, 1.051]

Table 9 shows the estimates of the fixed effects and intercept for the CALQ model. In both cases, the analyses highlighted a significant effect of the Author and Length variables on correctness, *p* = 0.021 and *p* = 0.004, respectively. An interaction between these two variables (Author × Length) was also observed, *p* = 0.002. The user-perceived quality of the stimuli had no significant effect on correctness.

**TABLE 9:** The estimated value, standard error, odds ratio, confidence interval, z-value, and *p*-value of the fixed effect for the CALQ model.

Effect	Estimate	Std. Error	Odds ratio	95% CI	z value	*p*-value
(intercept)	5.496	1.624	243.788	[2.577, 9.260]	3.384	< 0.001*
Author	−2.912	1.259	0.054	[−4.710, −0.477]	−2.312	0.021*
Length	−0.073	0.025	0.930	[−0.132, −0.028]	−2.871	0.004*
Quality Score	−0.129	0.187	0.879	[−0.504, 0.241]	−0.690	0.489
Author × Length	0.055	0.017	1.056	[0.081, 0.093]	3.153	0.002*
Author × Quality Score	−0.121	0.113	0.886	[−0.347, 0.102]	−1.067	0.286
Length × Quality Score	0.003	0.003	1.003	[−0.002, 0.009]	1.122	0.262

Note: * indicates a *p*-value below 0.05.

Since no significant effect of the Quality variable on correctness was observed, we considered it worthwhile to test whether this logistic regression model was better than the simple CA model. We conducted an analysis of deviance, which is a generalization of the residual sum of squares. The difference in deviance between the two models is asymptotically approximated to a *χ* ^2^ distribution. Therefore, a *p*-value below 0.05 indicates that two models are significantly different. The deviance calculation depends on the data via the maximum likelihood estimation method. The lower value on the Akaike Information Criterion (AIC) is an indicator of the better-suited model in the presence of a significant difference.

The results of the deviance analysis of CA vs. CALQ are shown in Table 10. The CALQ model was significantly better than the CA model (*χ* ^2^ = 17.570, *p* = 0.004), as indicated by the better goodness of fit. The lower AIC value of 897.007 indicates a better fit.

**TABLE 10:** Deviance analysis results: CA vs. CALQ.

Model	npar	AIC	logLik	Deviance	*χ* ^2^	df	*p*-value
CA	4	904.577	−448.289	896.578			
CALQ	9	897.007	−439.504	879.007	17.570	5	0.004*

Note: * indicates a *p*-value below 0.05.

It is conceivable that a model that excludes Quality but retains Length might be better than the saturated model in the presence of a non-significant difference between them. A model can be considered better if its AIC is lower and it includes fewer variables. Overall, we aim to adopt the most parsimonious model that provides the best explanation of the data. Therefore, the next step consisted of a comparison between the model represented in Eq. 4, which we refer to as CAL (Correctness–Author–Length), and the CALQ model.

Comparison of the CAL and CALQ models, as presented in Table 11, showed that there was no significant difference between them in terms of the goodness of fit.

**TABLE 11:** Deviance analysis results: CAL vs. CALQ.

Models	npar	AIC	logLik	Deviance	*χ* ^2^	df	*p* value
CAL	6	892.979	−440.490	880.979			
CALQ	9	897.007	−439.504	879.007	1.972	3	0.578

The Author–Length interaction model did not significantly change the goodness of fit compared to the saturated model, as indicated by *χ* ^2^ = 1.972, *p* = 0.578. However, the lower AIC score of 892.979 for the former model, combined with the model’s greater parsimony, implies that the CAL model was a better fit in explaining the data. The CAL model is described in Eq. 4.

$$\begin{array}{ll} & \text{Pr}\left(Y=\text{Correct}|y\right)=\frac{1}{1+e^{-y}}\\ & y=\text{Logit}\left(\frac{\text{Pr}\left(Y=\text{Correct}|\text{Author},\text{Length},{u_{0}}_{\text{PersonID}},{u_{0}}_{\text{StimulusPairID}}\right)}{1-\text{Pr}\left(Y=\text{Correct}|\text{Author},\text{Length},{u_{0}}_{\text{PersonID}},{u_{0}}_{\text{StimulusPairID}}\right)}\right)\\ & \;=\beta_{0}+\beta_{1}\times \text{Author}+\beta_{2}\times \text{Length}+\beta_{3}\times \text{Author}\times \text{Length}+{u_{0}}_{\text{PersonID}}+{u_{0}}_{\text{StimulusPairID}} \end{array}$$ (4)

Table 12 presents the variance and standard deviation of the random effects on the intercept of the CAL model.

**TABLE 12:** Variance, standard deviation, and confidence interval of the random effects for the CAL model.

Groups	Name	Variance	Std. Dev.	Std. Dev. 95% CI
PersonID	(Intercept)	0.426	0.653	[0.419, 0.950]
StimuliPairID	(Intercept)	0.174	0.417	[0.440, 1.086]

Table 13 shows the estimates of the fixed effects and intercept for the CAL model. All effects were statistically significant with *p* < 0.05.

**TABLE 13:** The estimated value, standard error, odds ratio, confidence interval, z-value, and *p*-value of the fixed effects for the CAL model.

Effect	Estimate	Std. Error	Odds ratio	95% CI	z value	*p*-value
(intercept)	5.141	1.391	170.849	[2.934, 8.588]	3.696	< 0.001*
Author	−3.814	0.986	0.022	[−5.932, −1.768]	−3.869	< 0.001*
Length	−0.062	0.023	0.939	[−0.120, −0.026]	−2.685	0.007*
Author ×Length	0.060	0.017	1.062	[ 0.028, 0.098]	3.460	< 0.001*

Note: * indicates a *p*-value below 0.05.

The results presented in Table 13 indicated a main effect of the Author and Length variables, as well as their interaction, on correctness. Moreover, the OR for Author (0.022) indicated that the odds of correct identification of the authorship of the stimuli decreased by 97.8% (95% CI [0.003, 0.152]) for AI-generated stimuli compared to human-generated stimuli.

The OR for Length (0.939) suggested that the odds of correct identification of the authorship of the stimuli decreased by 6.1% (95% CI [0.898, 0.983]) with each additional word. Finally, the OR for the Author–Length interaction (1.062) revealed that the odds of correct identification of the authorship of the stimuli increased by 6.2% (95% CI [1.027, 1.098]) for AI-generated stimuli compared to human-generated stimuli per additional word.

**FIGURE 6:**
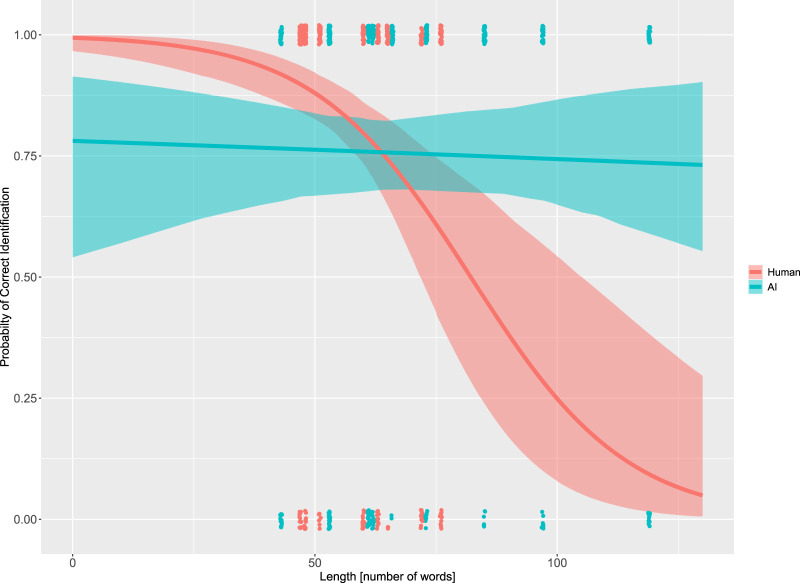
Stimulus length vs. probability of correct identification of authorship for human- vs. AI-generated stimuli.

To better understand these results, we created Figure 6. This illustrates the probability of correct identification of the authorship depending on the length of the stimulus and its authorship. This graph is slightly more complex than a simple line chart and requires a few explanatory words.

The *x*-axis represents the length of the stimuli in words. The *y*-axis represents the probability of correct identification. The red line shows the relationship between stimulus length and the probability of correct identification for the human-authored texts. This line is based on the CAL model, meaning that it represents an estimate. The light red boundaries indicate the confidence interval around the estimate.

Each red dot at the top and bottom represents a data point, meaning that we have 42 (participants) × 10 (texts) = 420 data points for human-generated stimuli. Each raw data point can only represent either a correct answer (1) or an incorrect answer (0). The raw data points therefore cannot be scatted across the probability scale. All data points would normally have to be concentrated at single points. To be able to see the various data points, we have slightly scatted them around their true values. This results in the point clouds observed on the top and bottom. The blue line, area, and points correspond to the same data for the AI-generated stimuli.

A visual inspection of the graphs indicates a slight negative slope for the blue line, which shows that the probability of correct identification of AI-generated text slowly decreases as the length of the text increases. The red line shows a much more dramatic change. It starts above the blue line and then rapidly falls. The swift decline means that the participants found it increasingly difficult to correctly identify the authorship of longer texts written by humans. The two lines cross at point [64, 0.757], which indicates an interaction effect between the length of the text and the author.

The graph illustrates the full logistic regression model. This means that it shows extrapolations for lines below a text length of 47 words and above 76 words for the human-generated texts. Notice that there are no data points below 47 words. There are also no red (human-generated text) data points above 76 words. Therefore, conclusions about lengths outside this range should be considered very preliminary.

#### 3.3.2 Question 5: how accurate and reliable are automatic AI detection systems?

Some AI detection systems could only be used partially or not at all in this study because they required a minimum number of characters of input that exceeded the length of our stimuli. TurnItIn required a minimum of 300 words and OpenAI 1,000 characters; none of our text stimuli were long enough to meet these criteria. GPTZero required a minimum of 250 characters, and hence could only be used with 5 of the human-generated stimuli. The analysis reported below was therefore conducted for 135 data points instead of the maximum of 140 with 7 × (10 + 10) = 140 (7 AI detectors, 10 human stimuli, 10 AI stimuli).

The AI detection systems were not consistent in their classification systems. Some of them reported a category (human or AI), while others provided continuous data, such as a percentage of the text that was AI-generated. We transformed all responses to the lowest common denominator, the categories of “human” and “AI”. For example, if an AI detection system provided a percentage of human authorship, then we categorized responses of 0–50 as “AI” and responses of 51–100 as “human”.

Table 14 shows the confusion matrix for the identifications by all the AI detection systems for the stimuli. The results show that AI detectors mainly identified the texts as being generated by humans, regardless of the actual authorship.

**TABLE 14:** Confusion matrix for the identifications by all the AI detection systems for all the stimuli.

		Identification		
		Human	AI	Total
Author	Human	TN 58 (89.2%)	FP 7 (10.8%)	65 (100.0%)
	AI	FN 70 (100.0%)	TP 0 (0.0%)	70 (100.0%)
	Total	128 (94.8%)	7 (5.2%)	135 (100.0%)

Notes: The values shown correspond to the identifications made by all seven AI detectors for the 20 stimuli. The total is 135 (140 − 5) due to five missing data points for human texts, as the number of words was less than the minimum required by one of the software packages. Percentages correspond to the value as a proportion of the total indicated in the cell on the corresponding row.

Table 15 shows the performance of each of the detectors.

**TABLE 15:** Number of correct identifications by each AI detection system.

	Author	
AI detection system	Human	AI
Undetectable.ai	9	0
GPTZero	*5	0
Copyleaks	10	0
Sapling	9	0
Contentatscale	8	0
ZeroGPT	8	0
HugginFace	9	0

Note: This table presents the number of correct identifications made by each of the seven AI detection systems for the human- and AI-generated stimuli; the values correspond to the TN and TP values, respectively, in Table 14. A higher number (maximum of 10) means more correct identifications, while a lower number (minimum of 0) means fewer correct identifications. *The maximum value for this cell is 5 because GPTZero, required a minimum number of characters that was not met by half of the stimuli.

We performed a *χ* ^2^ test to examine the association between the Author variable and the author predicted by automatic AI detection systems. Analyses revealed a significant association between the two variables, *χ* ^2^(1) = 7.95, *p* = 0.005. Fisher’s exact test supported these results (*p* = 0.005). However, the reported effect size was moderate, indicating a moderate association between the two variables (Cramer’s *V* = 0.243). With half of the stimuli generated by humans, the accuracy of the AI detection systems was 42.96%, slightly below chance level. The specificity of the automatic AI detection systems (89.23%) was above chance level. Recall and precision were null, as none of the detectors was able to correctly detect text generated by an AI. The F1 score could not be calculated, for the same reason.

**FIGURE 7:**
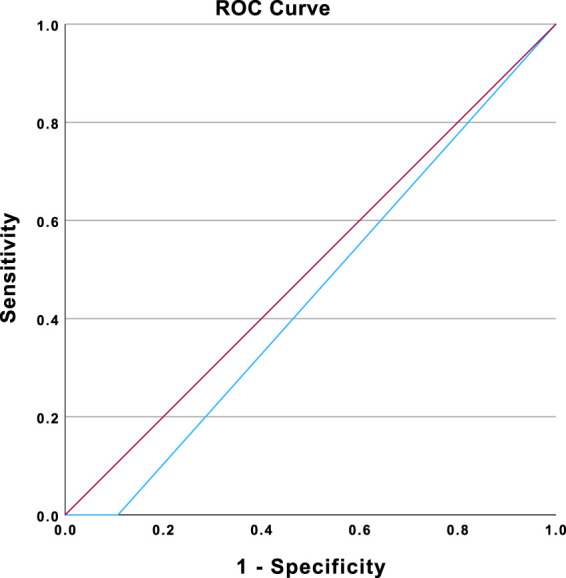
ROC curve representing the ability of the AI detection systems to detect AI-generated texts among AI- and human-generated texts. Note: The ROC curve is shown in blue. The diagonal reference line is shown in purple. Diagonal segments are produced by ties.

The ability of the systems to discriminate between AI and human texts was also analyzed using the area under the curve (AUC) (see Figure 7). The value of the AUC did not differ significantly from chance level (AUC = 0.446, *p* = 0.281). Therefore, the accuracy of the AI detection systems was no better than chance. The AI detection systems exhibited limited abilities to correctly detect authorship, at least for short texts generated by AI.

#### 3.3.3 Question 6: what is the relationship between the accuracy of AI detection systems and the accuracy of human participants?

A regression analysis was conducted to examine the correlation between the accuracy of the AI detection systems and the accuracy of the participants. A weak correlation between the two variables was observed, but this was not significant (*p* = 0.113). The accuracy of the human participants was not significantly correlated with that of the AI detection systems.

### 3.4 Criteria

#### 3.4.1 Question 7: what criteria are participants using to distinguish text generated by an AI from that written by a human?

Two coders were recruited to separately analyze the criteria given by the participants for making their choice in considering a text to have been written by a human or an AI. They were asked to create categories of factors that participants used to justify their choices and to place the justifications in these categories. Inter-coder reliability was very high, with a Cohen’s kappa of *κ* = 0.935, *p* < 0.001, indicating almost perfect agreement between both coders. After discussion between the two coders in order to revise the categorizations, perfect agreement was attained for all cases (*κ* = 1.0).

Participants reported using multiple ways of identifying whether the texts had been generated by humans or by AI. The most commonly reported factors, with the common justifications provided by participants, were as follows.

• **Text structure** (32 of the 42 participants). Several sub-categories were grouped together under this category. Participants considered texts to be AI-generated when they were long, with long sentences on a single subject. Punctuation errors indicated to participants that the text had been generated by a human. Structure in general was reported as a criterion, but with disagreement between participants: some considered texts to be AI-generated when they were well-structured, while others considered such texts to be human-generated. Tone was also considered as a factor influencing the choice of some participants, with a conversational tone considered more likely to be human-generated. Readability was also highlighted: if a text was difficult to read, it was thought to be AI-generated.
• **Vocabulary** (32/42). The use of overly technical, uncommon, or formal words led participants to believe that the author was an AI, while the use of informal words and slang (e.g., the term “lemon”) was an argument for considering the text to have been generated by a human. One participant also reported considering texts in which an abbreviation was generally used for a word (e.g., “automobile” in general becoming “auto”) to be more likely to have been written by an AI. Texts with a large vocabulary were also considered to be AI-generated.
• **Grammar and spelling errors** (22/42). Participants tended to consider spelling and grammatical errors as an argument in favor of the text having been generated by a human, as AIs would not make such mistakes.
• **Experience** (15/42). If the author drew on past experience, participants tended to consider the text to have been written by a human. Arguments based on facts were considered more likely to be AI-generated.
• **Provision of justifications** (12/42). There were several arguments that this feature was favored in AI-generated texts, with characteristics such as over-justifying by giving more information than necessary, or even providing definitions of terms, such that the reasoning failed to make sense. Fact-based justifications also fell into this category. One participant highlighted the fact that AI-generated texts seemed to use the same arguments every time.
• **Personal information** (8/42). Participants considered the author of a text to be human if they used the pronoun “I”, whereas they considered AI to tend to use “we” more generally. They also felt that the use of personal reasons was a factor influencing their choice. Some participants specified that the texts they considered to be AI-generated were more neutral than what a human would write.
• **Gut feeling** (6/42). Some participants said that they did not really have explicit knowledge of the factors influencing their choices, and rather responded based on instinct.
• **Emotions** (5/42). If the author used emotions, participants considered the text to be generated by a human.

#### 3.4.2 Question 8: is Undetectable.AI able to overcome AI detection systems?

We investigated the association between authorship and the author identified by automatic AI detection systems. For this analysis, the stimuli were the ChatGPT texts and the Undetectable.AI texts (abbreviated as UND). To do so, we performed a *χ* ^2^ test between the two variables. This revealed a significant association between the two variables, *χ* ^2^(1) = 7.37, *p* = 0.007. Fisher’s exact test supported these results (*p* = 0.013). The reported effect size was moderate, however, indicating a moderate association between the two variables, Cramer’s *V* = 0.229. The confusion matrix is shown in Table 16.

**TABLE 16:** Confusion matrix for the detection of AI authorship in Undetectable.AI- and ChatGPT-generated stimuli by AI detection systems.

		Detection		Total
		Human	AI	
Author	UND	TN 70 (50.0%)	FP 0 (0.0%)	70 (50%)
	ChatGPT	FN 63 (45.0%)	TP 7 (5.0%)	70 (50%)
	Total	133 (95.0%)	7 (5.0%)	140 (100.0%)

Notes. The values shown correspond to the identifications made by all seven AI detectors for the stimuli created by Undetectable.AI and ChatGPT. The total number of identifications made was 140. Percentages correspond to the value as a proportion of the total.

These results suggest that automatic AI detection systems mainly tended to attribute the stimuli to human authors. However, none of the stimuli obfuscated by Undetectable.AI were detected as being generated by an AI, whereas the automatic AI detection systems attributed a small percentage of ChatGPT-generated texts to AI. Thus, in our dataset, obfuscating sentences with Undetectable.AI was an effective solution for bypassing AI detectors.

## 4 Discussion

This research examined the extent to which LLMs like ChatGPT can create text that appears sufficiently similar to human-written text to fool researchers into thinking it was written by a human. The general aim of this study was to find out whether humans and automatic AI detection systems are able to distinguish AI-generated text from human-generated text in the context of online questionnaires. While some studies have addressed whether people can distinguish between texts written by humans and those written by LLMs in other contexts (Guo et al., 2023; Hämäläinen et al., 2023), to our knowledge, our study is the first to test this using LLMs and obfuscation services in the context of scientific research. Over the past 10–20 years, researchers have become more reliant on crowdsourcing sites to collect data from human participants. These sites, such as Mechanical Turk and Prolific, have many advantages over other forms of data collection, including the ability to collect large samples quickly and at low cost (Peer et al., 2017; Douglas et al., 2023). However, attention checks are important to guarantee high-quality responses. Until recently, the best practice was to ask participants to justify their answers in an open-ended response. While bots can answer multiple-choice and Likert-style questions easily, it has been expected that non-human responses to open-ended questions would be detected easily (Yarrish et al., 2019). However, LLMs may have changed the game, since these can discuss similar topics to humans (Hämäläinen et al., 2023). Bad actors can easily use LLMs and obfuscation services to participate in online studies to earn money. The current findings show that LLMs can generate responses that are difficult to detect as being AI-generated, meaning that researchers studying human responses may need to develop new ways of ensuring that responses to their questionnaires are actually written by humans.

### 4.1 Quality of the data and stimuli

The in-person nature of our study ensured that no bad actors could compromise the data collection with the use of bots or LLMs. The computers used were set to kiosk mode, preventing participants from leaving the questionnaire website. The data collected were of good quality according to various indicators, such as participants’ completion times, the coherence of their open-ended responses, and the absence of “straghtlining.” In addition, Qualtrics did not flag any participant as spamming. The stimuli were also similar, not only in terms of spelling and grammar mistakes but also in terms of length (i.e., number of words). The results showed that the generation process for the AI stimuli, which were based on the context in which the human stimuli were obtained, was capable of producing relevant representations of typical AI-generated text that were similar to those written by humans. Thus, it was necessary to check this similarity in terms of readability and user-perceived quality.

### 4.2 Readability and quality

The results of our analyses indicated that texts written by AI were more difficult to read. Indeed, the readability scores consistently placed the recommended reading ages for the human-generated texts below those of the AI-generated texts. The Undetectable.AI service does allow manipulation of the reading level. Its preset settings are: “high school,” “university,” “doctorate,” “journalist,” and “marketing.” The default value, which was used in our experiment, was “university.” The readability scores recorded seemed to support the claim of Undetectable.AI to generate text at a university level.

The readability scores did not significantly correlate with the participants’ ratings of the quality of the texts. While readability is certainly an important component of quality, it is possible that participants focused their attention more on the logic of the argument rather than on the rhetoric. We purposefully kept the user-perceived quality ratings abstract. It would have been possible to further divide quality into aspects of readability, such as grammar and spelling. However, this would have potentially excluded the more abstract “tone” of the text. Text generated by AI often feels like a Wikipedia article, which is different from personal and informal correspondence.

The user ratings of quality did not consistently differ between the texts written by humans and those generated by AI. The variation between the pairs of texts might be the reason for this lack of clear differences. While AI-generated sentences were slightly more often rated as being of higher quality, this might be based on the respective human-authored sentence being particularly informal and on the number of spelling and grammar mistakes. Indeed, even if these mistakes did not differ significantly between the human- and AI-generated groups of stimuli, participants might have detected the difference according to their magnitude. It seems that a difference of more than two mistakes influenced user ratings of quality. Taking this into account, it seems necessary to know how well humans and AI detection systems are able to correctly identify human-generated texts and AI-generated texts that have been created using an obfuscation system.

### 4.3 Imitation game

The results indicated that participants achieved an overall accuracy of 76% in identifying the authorship of texts. In contrast, AI detection systems did not perform any better than chance, with a bias toward identification of texts as human-generated. While some scholars have claimed that texts written by ChatGPT and other LLMs are indistinguishable from those written by humans (Susnjak, 2022; Lund et al., 2023), we show this is not entirely true. Studies aiming to see how well humans perform in identifying human- and AI-generated texts have obtained results more-or-less similar to ours (Gao et al., 2023; Guo et al., 2023; Nov et al., 2023). Hämäläinen et al. (2023) also used open-ended questions, but in a context of video games as art (while we have used these in a context of HRI), and observed lower identification accuracy than ours, with accuracy for AI-generated texts even below the level of chance. We might say first that this is surprising, since they used longer texts and we might have expected that this would provide more information for people and therefore improve their accuracy in identification of authorship. However, our results do not support this and seem to provide an explanation of the lower accuracy reported in Hämäläinen et al. (2023), as the odds of correctly identifying the authorship of a stimulus decreased by 6.1% for each additional word, rather than increasing. However, the research methodologies used in other papers are quite different, such as the use of longer texts and different participant groups [e.g., experts and non-experts in Guo et al. (2023)], which makes direct comparisons difficult. Still, the results seem to point roughly in the same direction. It would be desirable to develop a standardized research method that would allow us to reliably replicate the results of others.

Automatic AI detection systems perform much worse than humans. It is therefore not surprising that their performance did not significantly correlate with that of humans. Some companies have started to openly admit to the poor performance of their systems. OpenAI took its AI classifier offline due to its low accuracy as of 20 July 2023, a few weeks after we completed our data collection. It is interesting to see that OpenAI reported that their classifier detects only 26% of AI-written text.^21^ Their honesty about the weaknesses of their automatic AI detection system is noteworthy, given that other AI detection systems emphasize the strong capabilities of their software. It is not unlikely, therefore, that the detectors have improved since this paper was written, as have the capabilities of the LLMs. This will remain a cat-and-mouse game.

Since humans perform so much better than automatic AI detection systems, it is worth investigating how humans achieve this. Participants reported making their choices using several criteria, such as text structure (32/42); vocabulary (32/42); typos, grammar, and spelling mistakes (22/42); use of experience (15/42), emotions (5/42), or personal information (8/42) in the texts; and the arguments used in the texts (12/42). Six participants reported using their instincts and not really knowing how they made their choices. These results are consistent with those of Kumarage et al. (2023), who used stylometry (i.e., phraseology, punctuation, and linguistic diversity) to detect AI-generated texts. Phraseology and punctuation correspond to our vocabulary and text structure categories. Linguistic diversity analysis was conducted using the Flesch readability score. The criteria used by our participants are consistent with those reported in other research, such as considering a text to be human-generated when emotional experiences are involved (Guo et al., 2023; Hämäläinen et al., 2023), and considering a text to be AI-generated when the vocabulary used is atypical, objective and neutral, formal, impersonal, structured, and verbose (Borji, 2023; Guo et al., 2023; Mitrovic et al., 2023). It is conceivable that participants in this study had already used ChatGPT and used their knowledge of it as a baseline in making their decisions. Interestingly, while data analyses did not highlight significant differences between ChatGPT and Undetectable.AI stimuli in terms of length or spelling and grammar mistakes, participants claimed to have used these criteria. This discrepancy between self-reports and behavioral observations is not uncommon. At this point in time, it is unclear whether implicit measurements are necessary to better understand how participants identify authorship.

While humans seem to perform better with shorter texts, automatic AI detection systems perform better with longer texts. As a consequence, it would be advisable to set a maximum text entry length when humans will be used to identify authorship. If automatic AI detection systems will be used, then a minimum text entry length should be enforced. Unfortunately, these two requirements contradict each other. A possible workaround would be to have two text entries in an online form: one that will be judged by humans and a second that will be judged by automatic AI detection systems.

### 4.4 Undetectable.AI

Undetectable.AI and similar services that are likely to spawn in the near future present a considerable challenge. While other AI-supported writing tools, such as Quillbot^22^ and Jenni,^23^ focus on assisting the writing process, Undetectable.AI clearly markets itself as an obfuscation tool. On their website they indicate directly which automatic AI detection systems will be overcome. We compared the accuracy of authorship detection for text written by Undetectable.AI to that of text written by ChatGPT directly. Automatic AI detection systems identified ChatGPT stimuli as AI-generated 10% of the time, whereas none of the same stimuli humanized using Undetectable.AI were identified as AI-generated. The false-negative rate was therefore 90% for ChatGPT and 100% for Undetectable.AI. This result aligns with the work of Mitrovic et al. (2023), who showed that rephrasing makes ChatGPT texts more difficult to detect. While it is largely unclear how Undetecable.AI works, we were able to observe that it does add spelling and grammar mistakes to the text. Their frequency was similar to that seen in text written by humans.

AI text generators and detection systems will likely continue to play a cat-and-mouse game. This is somewhat similar to the generation of spam emails. Both sides will continue to train their system to defeat the other. At this point in time, the AI generators are ahead in the game, making it nearly impossible to automatically detect their use, particularly in shorter passages of text. Competitors to Undetectable.AI are likely to emerge in the near future. While their business model is based on deception, it is unlikely that they could be shut down through the courts; even if this were to be possible, new services would continue to spawn. Pandora’s box has been opened and society will have to learn how to utilize these new tools for the benefit of all.

## 5 Conclusion and future work

The results of our experiment show that the arrival of LLM services, such as Undetectable.AI, renders automatic AI detection systems ineffective. These systems produce far too many false negatives and thereby allow AI-generated responses into data analysis. This has the potential to fundamentally corrupt data collected in online questionnaires. Moreover, the responses to open questions collected in online questionnaires do not often meet the minimum length required to enable any chance at effective automatic AI detection.

One could argue that asking participants in online studies to write more text might be a solution, since this might enable better performance by automatic AI detection systems. However, this idea fails to recognise the power of obfuscation services, such as Undetectable.AI. Moreover, since participants are paid according to the amount of time they spend on a questionnaire and additional length comes at no practical cost for LLMs, experimenters will only set up more attractive targets for abuse. Having to write long text answers might also discourage participants from completing an experiment and might introduce a bias towards participants with high levels of literacy.

The participants in our experiment were students who were familiar with computer science. Their overall accuracy in identifying the authorship of texts was approximately 76%. While this is significantly above chance level, it is not as accurate as one might hope, given the normally accepted level of false positives in psychological experiments of 5%. Guo et al. (2023) showed that when humans have to distinguish between texts of AI origin and human origin, if the texts are presented one by one, performance is lower than when the texts are presented in pairs. This strategy could be adopted by experimenters to increase performance in identification.

It remains to be seen how many bots using LLMs will start to plague online experiments. In our study, we used a 50% proportion of AI-generated stimuli. This aligns with the proportion of spam emails in recent years. Griffin et al. (2022) estimated that 27.4% of their 709 responses were possibly from bots. We have no reason to believe that bad actors would not seek out this opportunity to earn money. It is conceivable that bad actors could easily target all online questionnaires posted on crowdsourcing platforms. It is therefore possible that almost all responses collected would be generated by bots using LLMs. If, for example, 90% of all submissions to an online questionnaire are not from humans, then even using humans to filter these out will still not yield enough usable data.

**FIGURE 8:**
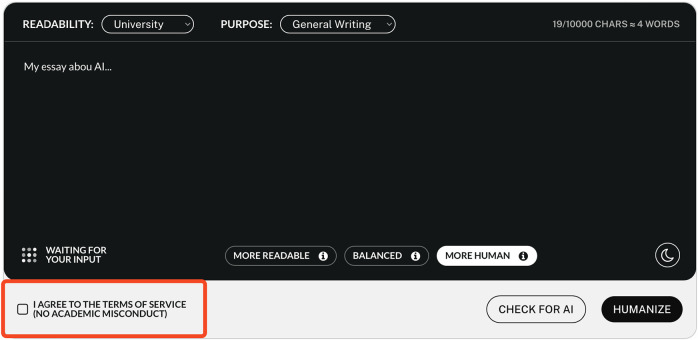
The Undetectable.AI interface requires the acceptance of their terms of service. The red box has been added by the authors to highlight the requirement to agree to the terms of service.

There is no reason to believe that bad actors would refrain from using services such as Undetectable.AI. While this service is far less known in general compared to ChatGPT or Bard, it fills an important niche. Before executing any transformation, Undetectable.AI asks the user to affirm that he or she will not engage in any academic misconduct (see Figure 8). By asking users not to engage in academic misconduct, they do of course point out that it is possible to use the service to do so. Again, because this is a systematic risk and individual scholars cannot reliably eliminate all AI responses from their own data, the focus is on providers of these technologies to minimize their use for scientific misconduct. Asking users not to engage in this is one step, but other consequences may become necessary if too many users violate this rule.

Unless the crowdsourcing platforms acknowledge this problem and prevent payment for bot submissions, the economic incentive to abuse the system will remain. Some crowdsourcing platforms allow researchers to deny payment for unacceptable submissions. However, this verification process is labor-intensive and can be (automatically) challenged by the participant. It would be desirable if the crowdsourcing platforms would take a more active role in the prevention of fraud by fundamentally challenging the business model of bots.

Services like Prolific have some quality controls and achieve relatively low rates of poor-quality data. If the percentage of bots is lower, and humans are able to accurately detect them approximately 76% of the time, this would not be a major problem. The key, then, is to ensure high rates of quality participants on the crowdsourcing platforms. AI will threaten researchers’ abilities to identify individual bad actors, so the solution has to be systematic. A bad actor might be able to use AI to generate bad responses and get away with it sometimes, but if crowdsourcing platforms are vigilant in identifying accounts using bots and eliminating their responses approximately three-quarters of the time, bad actors will struggle to prosper.

At this point, almost all methods of automatically distinguishing humans from bots and LLMs have failed in one way or another. Bots have become faster and more accurate at solving CAPTCHAs (Searles et al., 2023). Increasing the difficulty of these is not an option, since this would make it too difficult for humans to solve them. Neither CAPTCHAs nor free text responses offer sufficient protection. Researchers and platforms have the option to increase their efforts by combining various methods, which is our recommendation. However, this will eventually lead to diminishing returns. At some point, conducting questionnaires online may no longer be quicker and cheaper than conducting them in person.

This concern applies not only to the field of HRI, but to any segment of the wider research community that uses online questionnaires. Problems with data quality will only ever increase the difficulties in conducting studies that can be reliably replicated. The concerns raised in this study thereby fundamentally apply to the replication crisis and its possible remedies.

### 5.1 Implications for education

Not only do the challenges of LLMs apply to experiments in HRI, but they also have the potential to impact many aspects of education. Students can use LLMs to generate responses to quizzes and even to write entire essays or cheat on their exams (Susnjak, 2022; Rahman and Watanobe, 2023). Some LLMs have been able to pass academic examinations in various areas, such as computer science (Bordt and von Luxburg, 2023) or medicine (Gilson et al., 2022; Kung et al., 2023). This has led some academics to develop concerns about classic assessment methods and the impact on critical thinking, while others believe that we will still be able to identify AI-generated essays because of the possible poor capabilities of the AI services, which potentially provide incorrect or irrelevant information.^24^ However, people might blindly rely on ChatGPT (Rahman and Watanobe, 2023).

Plagiarism software providers, such as TurnItIn, have recently announced that their software can now detect content generated by an LLM.^25^ While some scholars have attempted to examine the performance of these AI detectors (see Uzun (2023) for a non-systematic list of some current AI detectors), their accuracy remains insufficiently clear. Our data suggest that, at least in the case of short answers to prompts, human readers may be better able to detect AI responses than automated detectors. However, their accuracy level may still not be sufficient to prove academic misconduct to a sufficient standard for disciplinary action to be taken.

### 5.2 Limitations and future work

It would have been possible to conduct this experiment completely online using one of the popular platforms to recruit participants and conduct the questionnaire. While this would have allowed us to conveniently increase the number of participants, it would have had the potential to be manipulated by bad actors. To ensure that no AI was participating in our study, we decided to conduct it in person. We are aware that the convenience sampling employed has its own limitations.

Some details relating to the participants need to be discussed. While a sample size of 42 participants does not seem to be much greater according to what was discussed in the introduction (Baxter et al., 2016), G*Power was used *a priori* and indicated a required minimum sample size of 31 participants for two-tailed logistic regression analyses, based on the odds ratio collected from the results of Gao et al. (2023), an expected power of 0.8, and alpha of 0.05. Using one-tailed logistic regression analyses did not lead to an underpowered study, as a minimum of 40 participants was required using the same G*Power settings. It is also important to note that although Gao et al. (2023) did not use a within-participants design (their participants were each exposed to half of the stimuli), our study did, with 20 stimuli. It would therefore be expected to achieve sufficient power, if not more, with fewer participants. Most of the participants in our study had a technical background. Students from different disciplines might be less capable of identifying an AI system. Guo et al. (2023) showed that people who frequently use ChatGPT achieve better accuracy in identifying its output than people who have never heard about ChatGPT. It would therefore be desirable to replicate this study with wider sampling. It is also not a given that computer science students will always be better at detecting AI-generated text. It is conceivable that students in other fields, such as English literature, might be more fine-tuned to the style and voice of text and therefore better at detecting AI-generated text.

We do not have any evidence on how many responses to online questionnaires are currently being created by AI; nor can we accurately predict how prevalent this problem will become. What is certain is that it typically only takes a small number of bad actors to erode trust in the common good. Honesty boxes normally disappear quickly if small numbers of criminals can take advantage of them.

## Data Availability

All data (raw and data analysis) supporting the conclusion of this article can be found at http://doi.org/10.17605/OSF.IO/TFA48.

## Footnotes

1
https://www.qualtrics.com/support/survey-platform/survey-module/survey-checker/response-quality

2
https://openai.com/blog/chatgpt

3 ChatGPT based on OpenAI’s GPT-4 architecture; for ease of reading, “ChatGPT” will be followed by the number of the GPT model on which it is based.

4
https://nypost.com/2023/03/17/the-manipulative-way-chatgpt-gamed-the-captcha-test/

5
https://github.com/Significant-Gravitas/Auto-GPT

6
https://www.qualtrics.com

7 Version 3.5 as of June 2023: http://chat.openai.com

8
https://undetectable.ai

9
https://www.linguisticanalysistools.org/arte.html

10
http://gunning-fog-index.com/index.html

11
https://www.grammarly.com

12
https://undetectable.ai

13
https://gptzero.me

14
https://copyleaks.com/ai-content-detector

15
https://sapling.ai/ai-content-detector

16
https://contentatscale.ai/ai-content-detector/

17
https://www.zerogpt.com

18
https://ai-content-detector.online or huggingface.co/spaces/PirateXX/AI-Content-Detector

19
https://originality.ai

20
https://platform.openai.com/ai-text-classifier

21
https://openai.com/blog/new-ai-classifier-for-indicating-ai-written-text

22
https://quillbot.com

23
https://jenni.ai

24
https://www.nature.com/articles/d41586-022-04397-7

25
https://www.turnitin.com/blog/the-launch-of-turnitins-ai-writing-detector-and-the-road-ahead

## References

[B1] Adams T. L. Li Y. Liu H. (2020). A replication of beyond the turk: alternative platforms for crowdsourcing behavioral research–sometimes preferable to student groups. AIS Trans. Replication Res. 6, 15. 10.17705/1atrr.00058

[B2] Ahn L. v. Blum M. Hopper N. J. Langford J. (2003). “Captcha: using hard ai problems for security,” in International conference on the theory and applications of cryptographic techniques (Springer), 294–311. 10.1007/3-540-39200-9_18

[B3] Argyle L. P. Busby E. C. Fulda N. Gubler J. R. Rytting C. Wingate D. (2023). Out of one, many: using language models to simulate human samples. Polit. Anal. 31, 337–351. 10.1017/pan.2023.2

[B4] Bainbridge W. A. Hart J. W. Kim E. S. Scassellati B. (2011). The benefits of interactions with physically present robots over video-displayed agents. Int. J. Soc. Robotics 3, 41–52. 10.1007/s12369-010-0082-7

[B5] Baker M. (2016). 1,500 scientists lift the lid on reproducibility. Nat. News 533, 452–454. 10.1038/533452a 27225100

[B6] Bartneck C. Duenser A. Moltchanova E. Zawieska K. (2015). Comparing the similarity of responses received from studies in amazon’s mechanical turk to studies conducted online and with direct recruitment. PLOS ONE 10, 1–23. 10.1371/journal.pone.0121595 PMC4397064 25876027

[B7] Baumeister R. F. (2016). Charting the future of social psychology on stormy seas: winners, losers, and recommendations. J. Exp. Soc. Psychol. 66, 153–158. Rigorous and replicable methods in social psychology. 10.1016/j.jesp.2016.02.003

[B8] Baxter P. Kennedy J. Senft E. Lemaignan S. Belpaeme T. (2016). “From characterising three years of hri to methodology and reporting recommendations,” in The eleventh ACM/IEEE international conference on human robot interaction (IEEE press), HRI ’16, 391–398. 10.1109/HRI.2016.7451777

[B9] Belhassein K. Buisan G. Clodic A. Alami R. (2019). “Towards methodological principles for user studies in human-robot interaction,” in Test methods and metrics for effective HRI in collaborative human-robot teams workshop (ACM/IEEE International Conference on Human-Robot Interaction), 1–7.

[B10] Belpaeme T. (2020). Advice to new human-robot interaction researchers, 12. Cham: Springer International Publishing, 355–369. 10.1007/978-3-030-42307-0_14

[B11] Blanca M. J. Alarcon R. Arnau J. Bono R. Bendayan R. (2017). Non-normal data: is anova still a valid option? Psicothema 29, 552–557. 10.7334/psicothema2016.383 29048317

[B12] Bordt S. von Luxburg U. (2023). Chatgpt participates in a computer science exam. *arXiv* doi. 10.48550/arXiv.2303.09461

[B13] Borji A. (2023). A categorical archive of chatgpt failures. arXiv. 10.48550/arXiv.2302.03494

[B14] Buchanan E. M. Scofield J. E. (2018). Methods to detect low quality data and its implication for psychological research. Behav. Res. Methods 50, 2586–2596. 10.3758/S13428-018-1035-6 29542063

[B15] Buhrmester M. Kwang T. Gosling S. (2011). Amazon’s mechanical turk: a new source of inexpensive, yet high-quality, data? Perspect. Psychol. Sci. 6, 3–5. 10.1177/1745691610393980 26162106

[B16] Chall J. S. Dale E. (1995). Readability revisited: the new Dale-Chall readability formula. Cambridge, Mass: Brookline Books.

[B17] Copeland J. Proudfoot D. (2009). “Turing’s test: a philosophical and historical guide,” in Parsing the turing test: philosophical and methodological issues in the quest for the thinking computer. Editors Epstein G. Roberts R. Beber G. (Dordrecht: Springer), 119–138. Export Date: 10 July 2022; Cited By: 14. 10.1007/978-1-4020-6710-5_9

[B18] Daniel F. Kucherbaev P. Cappiello C. Benatallah B. Allahbakhsh M. (2018). Quality control in crowdsourcing: a survey of quality attributes, assessment techniques, and assurance actions. ACM Comput. Surv. 51, 1–40. 10.1145/3148148

[B19] Das M. Cui R. (2019). Comparison of quality indicators in user-generated content using social media and scholarly text. *arXiv* . 10.48550/arXiv.1910.11399

[B20] Devlin J. Chang M.-W. Lee K. Toutanova K. (2019). Bert: pre-training of deep bidirectional transformers for language understanding. *arXiv* . 10.48550/arXiv.1810.04805

[B21] Douglas B. D. Ewell P. J. Brauer M. (2023). Data quality in online human-subjects research: comparisons between mturk, prolific, cloudresearch, qualtrics, and sona. PLOS ONE 18, 1–17. 10.1371/journal.pone.0279720 PMC10013894 36917576

[B22] Dubay W. H. (2004). The principles of readability. CA. Costa Mesa: Impact Information. 92627949, 631–3309.

[B23] DuBay W. H. (2007). *Smart language: readers, readability, and the grading of text* (costa mesa, calif.: impact information).

[B24] Feigenbaum E. A. (2003). Some challenges and grand challenges for computational intelligence. J. ACM (JACM) 50, 32–40. 10.1145/602382.602400

[B25] Flesch R. (1948). A new readability yardstick. J. Appl. Psychol. 32, p221–p233. 10.1037/h0057532 18867058

[B26] Gamblin B. Winslow M. Lindsay B. Newsom A. Kehn A. (2017). Comparing in-person, sona, and mechanical turk measurements of three prejudice-relevant constructs. Curr. Psychol. 36, 217–224. 10.1007/s12144-015-9403-1

[B27] Gao C. A. Howard F. M. Markov N. S. Dyer E. C. Ramesh S. Luo Y. (2023). Comparing scientific abstracts generated by chatgpt to real abstracts with detectors and blinded human reviewers. npj Digit. Med. 6, 75. 10.1038/s41746-023-00819-6 37100871 PMC10133283

[B28] Gilson A. Safranek C. Huang T. Socrates V. Chi L. Taylor R. A. (2022). How does chatgpt perform on the medical licensing exams? the implications of large language models for medical education and knowledge assessment. medRxiv. 10.1101/2022.12.23.22283901 PMC9947764 36753318

[B29] Godinho A. Schell C. Cunningham J. A. (2020). Out damn bot, out: recruiting real people into substance use studies on the internet. Subst. Abuse 41, 3–5. 10.1080/08897077.2019.1691131 31821108

[B30] Gomez Adorno H. Rios G. Posadas Durán J. Sidorov G. Sierra G. (2018). Stylometry-based approach for detecting writing style changes in literary texts. Comput. Sist. 22. 10.13053/cys-22-1-2882

[B31] Griffin M. Martino R. J. LoSchiavo C. Comer-Carruthers C. Krause K. D. Stults C. B. (2022). Ensuring survey research data integrity in the era of internet bots. Qual. Quantity 56, 2841–2852. 10.1007/s11135-021-01252-1 PMC8490963 34629553

[B32] Gunning R. (1952). The technique of clear writing. McGraw-Hill.

[B33] Guo B. Zhang X. Wang Z. Jiang M. Nie J. Ding Y. (2023). How close is chatgpt to human experts? comparison corpus, evaluation, and detection. *arXiv* . 10.48550/arXiv.2301.07597

[B34] Hämäläinen P. Tavast M. Kunnari A. (2023). Evaluating large language models in generating synthetic hci research data: a case study. CHI ’23: CHI conference on human factors in computing systems. (New York, NY, United States; ACM), 1–19. 10.1145/3544548.3580688

[B35] Hamby T. Taylor W. (2016). Survey satisficing inflates reliability and validity measures: an experimental comparison of college and amazon mechanical turk samples. Educ. Psychol. Meas. 76, 912–932. 10.1177/0013164415627349 29795893 PMC5965608

[B36] Irfan B. Kennedy J. Lemaignan S. Papadopoulos F. Senft E. Belpaeme T. (2018). Social psychology and human-robot interaction: an uneasy marriage. Companion of the 2018 ACM/IEEE international conference on human-robot interaction, HRI’18. New York, NY, USA: Association for Computing Machinery, 13–20. 10.1145/3173386.3173389

[B37] Kerr N. (1998). Harking: hypothesizing after the results are known. Personality Soc. Psychol. Rev. official J. Soc. Personality Soc. Psychol. Inc 2, 196–217. 10.1207/s15327957pspr0203_4 15647155

[B38] Kincaid J. P. Fishburne R. P. Rogers R. L. Chissom B. S. (1975). Derivation of new readability formulas (automated readability index, fog count and flesch reading ease formula) for navy enlisted personnel. Institute for Simulation and Training, University of Central Florida.

[B39] Krosnick J. A. (1991). Response strategies for coping with the cognitive demands of attitude measures in surveys. Appl. Cogn. Psychol. 5, 213–236. 10.1002/acp.2350050305

[B40] Kuek S. C. Paradi-Guilford C. Fayomi T. Imaizumi S. Ipeirotis P. Pina P. (2015). The global opportunity in online outsourcing. Washington DC, United States: World Bank Publications - Reports 22284, The World Bank Group.

[B41] Kumarage T. Garland J. Bhattacharjee A. Trapeznikov K. Ruston S. Liu H. (2023). Stylometric detection of ai-generated text in twitter timelines. *arXiv* doi. 10.48550/arXiv.2303.03697

[B42] Kung T. H. Cheatham M. Medenilla A. Sillos C. De Leon L. Elepaño C. (2023). Performance of chatgpt on usmle: potential for ai-assisted medical education using large language models. PLOS Digit. Health 2, e0000198. 10.1371/journal.pdig.0000198 36812645 PMC9931230

[B43] Leichtmann B. Nitsch V. (2020a). How much distance do humans keep toward robots? literature review, meta-analysis, and theoretical considerations on personal space in human-robot interaction. J. Environ. Psychol. 68, 101386. 10.1016/j.jenvp.2019.101386

[B44] Leichtmann B. Nitsch V. (2020b). Is the social desirability effect in human–robot interaction overestimated? a conceptual replication study indicates less robust effects. Int. J. Soc. Robotics 13, 1013–1031. 10.1007/s12369-020-00688-z

[B45] Leichtmann B. Nitsch V. Mara M. (2022). Crisis ahead? why human-robot interaction user studies may have replicability problems and directions for improvement. Front. Robotics AI 9, 838116. 10.3389/frobt.2022.838116 PMC8961736 35360497

[B46] Li J. (2015). The benefit of being physically present: a survey of experimental works comparing copresent robots, telepresent robots and virtual agents. Int. J. Human-Computer Stud. 77, 23–37. 10.1016/j.ijhcs.2015.01.001

[B47] Lund B. Wang T. Mannuru N. R. Nie B. Shimray S. Wang Z. (2023). Chatgpt and a new academic reality: artificial intelligence-written research papers and the ethics of the large language models in scholarly publishing. J. Assoc. Inf. Sci. Technol. 74, 570–581. 10.1002/asi.24750

[B48] Mao A. Kamar E. Chen Y. Horvitz E. Schwamb M. Lintott C. (2013). Volunteering versus work for pay: incentives and tradeoffs in crowdsourcing. Proc. AAAI Conf. Hum. Comput. Crowdsourcing 1, 94–102. 10.1609/hcomp.v1i1.13075

[B49] Mc Laughlin G. H. (1969). Smog grading-a new readability formula. J. Read. 12, 639–646.

[B50] Mitrovic S. Andreoletti D. Ayoub O. (2023). Chatgpt or human? detect and explain. explaining decisions of machine learning model for detecting short chatgpt-generated text. *arXiv* . 10.48550/arXiv.2301.13852

[B51] Moor J. H. (2001). The status and future of the turing test. Minds Mach. 11, 77–93. 10.1023/A:1011218925467

[B52] Naglieri J. Drasgow F. Schmit M. Handler L. Prifitera A. Margolis A. (2004). Psychological testing on the internet: new problems, old issues. Am. Psychol. 59, 150–162. 10.1037/0003-066X.59.3.150 15222858

[B53] Nov O. Singh N. Mann D. (2023). Putting chatgpt’s medical advice to the (turing) test. medRxiv. 10.1101/2023.01.23.23284735 PMC10366957 37428540

[B54] Oliveira R. Arriaga P. Santos F. Mascarenhas S. Paiva A. (2020). Towards prosocial design: a scoping review of the use of robots and virtual agents to trigger prosocial behaviour. Comput. Hum. Behav. 114, 106547. 10.1016/j.chb.2020.106547

[B55] Open Science Collaboration (2015). PSYCHOLOGY. Estimating the reproducibility of psychological science. Science 349, aac4716. 10.1126/science.aac4716 26315443

[B56] Peer E. Brandimarte L. Samat S. Acquisti A. (2017). Beyond the turk: alternative platforms for crowdsourcing behavioral research. J. Exp. Soc. Psychol. 70, 153–163. 10.1016/j.jesp.2017.01.006

[B57] Peng R. D. (2011). Reproducible research in computational science. Science 334, 1226–1227. 10.1126/science.1213847 22144613 PMC3383002

[B58] Powers A. Kiesler S. Fussell S. Torrey C. (2007). “Comparing a computer agent with a humanoid robot,” in Proceedings of the ACM/IEEE international conference on human-robot interaction (New York, NY, USA: Association for Computing Machinery), HRI ’07), 145–152. 10.1145/1228716.1228736

[B59] Pozzar R. Hammer M. J. Underhill-Blazey M. Wright A. A. Tulsky J. A. Hong F. (2020). Threats of bots and other bad actors to data quality following research participant recruitment through social media: cross-sectional questionnaire. J. Med. Internet Res. 22, e23021. 10.2196/23021 33026360 PMC7578815

[B60] Rahman M. M. Watanobe Y. (2023). Chatgpt for education and research: opportunities, threats, and strategies. Appl. Sci. 13, 5783. 10.3390/app13095783

[B61] Rudolph J. Tan S. Tan S. (2023). Chatgpt: bullshit spewer or the end of traditional assessments in higher education? J. Appl. Learn. Teach. 6. 10.37074/jalt.2023.6.1.9

[B62] Searles A. Nakatsuka Y. Ozturk E. Paverd A. Tsudik G. Enkoji A. (2023). An empirical study and evaluation of modern captchas. *arXiv* . 10.48550/arXiv.2307.12108

[B63] Si L. Callan J. (2001). “A statistical model for scientific readability,” in Proceedings of the tenth international conference on information and knowledge management (New York, NY, USA: Association for Computing Machinery), 574–576. CIKM ’01. 10.1145/502585.502695

[B64] Simmons J. P. Nelson L. D. Simonsohn U. (2011). False-positive psychology: undisclosed flexibility in data collection and analysis allows presenting anything as significant. Psychol. Sci. 22, 1359–1366. 10.1177/0956797611417632 22006061

[B65] Smith E. A. Senter R. J. (1967). Automated readability index. Amrl Tr., 1–14. 5302480

[B66] Strait M. Lier F. Bernotat J. Wachsmuth S. Eyssel F. Goldstone R. (2020). “A three-site reproduction of the joint simon effect with the nao robot,” in Proceedings of the 2020 ACM/IEEE international conference on human-robot interaction (New York, NY, USA: Association for Computing Machinery), 103–111. HRI ’20. 10.1145/3319502.3374783

[B67] Susnjak T. (2022). Chatgpt: the end of online exam integrity? *arXiv* . 10.48550/arXiv.2212.09292

[B68] Swiatkowski W. Dompnier B. (2017). Replicability crisis in social psychology: looking at the past to find new pathways for the future. Int. Rev. Soc. Psychol. 30, 111–124. 10.5334/irsp.66

[B69] Teitcher J. E. Bockting W. O. Bauermeister J. A. Hoefer C. J. Miner M. H. Klitzman R. L. (2015). Detecting, preventing, and responding to “fraudsters” in internet research: ethics and tradeoffs. J. Law Med. Ethics 43, 116–133. 10.1111/jlme.12200 25846043 PMC4669957

[B70] Tenney E. R. Costa E. Allard A. Vazire S. (2021). Open science and reform practices in organizational behavior research over time (2011 to 2019). Organ. Behav. Hum. Decis. Process. 162, 218–223. 10.1016/j.obhdp.2020.10.015.

[B71] Thellman S. Silvervarg A. Gulz A. Ziemke T. (2016). Physical vs. virtual agent embodiment and effects on social interaction. Int. Conf. Intelligent Virtual Agents 10011, 412–415. 10.1007/978-3-319-47665-0_44

[B72] Touvron H. Lavril T. Izacard G. Martinet X. Lachaux M.-A. Lacroix T. (2023). Llama: open and efficient foundation language models. *arXiv* . 10.48550/arXiv.2302.13971

[B73] Turing A. Braithwaite R. Jefferson G. Newman M. (2004). “Can automatic calculating machines be said to think? (1952),” in The essential turing. Editor Copeland J. (Clarendon Press), 487–515.

[B74] Turing A. M. (1950). I.—COMPUTING MACHINERY AND INTELLIGENCE. Mind LIX, 433–460. 10.1093/mind/LIX.236.433

[B75] Turing A. M. (2004). “Intelligent machinery,” in The essential turing. Editor Copeland J. (Clarendon Press, National Physical Laboratory Report), 395–432.

[B76] Ullman D. Aladia S. Malle B. F. (2021). “Challenges and opportunities for replication science in hri: a case study in human-robot trust,” in Proceedings of the 2021 ACM/IEEE international conference on human-robot interaction (New York, NY, USA: Association for Computing Machinery), 110–118. HRI ’21. 10.1145/3434073.3444652

[B77] Ullman D. Malle B. F. (2017). Human-robot trust: just a button press away. Proceedings of the companion of the 2017 ACM/IEEE international conference on human-robot interaction (New York, NY, USA: Association for Computing Machinery), 309–310. HRI ’17. 10.1145/3029798.3038423

[B78] Uzun L. (2023). Chatgpt and academic integrity concerns: detecting artificial intelligence generated content. Lang. Educ. Technol. 3, 45–54.

[B79] Vonasch A. Mofradidoost R. Gray K. (2022). When people reject free money: phantom costs and the psychology of economic exchange. PsyArXiv. 10.31234/osf.io/fcery PMC12361704 38587190

[B80] Wu M.-J. Zhao K. Fils-Aime F. (2022). Response rates of online surveys in published research: a meta-analysis. Comput. Hum. Behav. Rep. 7, 100206. 10.1016/j.chbr.2022.100206

[B81] Yarrish C. Groshon L. Mitchell J. D. Appelbaum A. Klock S. Winternitz T. (2019). Finding the signal in the noise: minimizing responses from bots and inattentive humans in online research. Behav. Ther. 42, 235–242.

