# Supplementary material for: Detecting the corruption of online questionnaires by artificial intelligence
Source: Front Robot AI. 2024 Feb 2;10:1277635. doi: 10.3389/frobt.2023.1277635 (PMC10869497; doi:10.3389/frobt.2023.1277635)
Supplement: Supplementary file 1 [file DataSheet1.pdf]

APPENDIX A: TEXT GENERATION PROTOCOL

We used the following protocol to generate text using AI systems:

1. Prompt ChatGPT to play the role of a participant participating in a research study.
2. Prompt ChatGPT to not provide more information than necessary and to not start its sentence by saying its role.
3. Provide the context of the study to ChatGPT as if it was a real participant (i.e., information letter and consent form).
4. Provide the context used in the source study along with the same question participants had to answer. Ask ChatGPT to provide a response for each item on the 7-point Likert scale from strongly disagree to strongly agree.
5. Ask ChatGPT to elaborate the response of the item of interest (e.g., strongly disagree) with a word count similar to the paired human sentence. If good, go to step 7.
6. If the number of words is highly different and/or the text is not original, generate a new answer up to five times. If five answers were generated and are still different, take the one with the closest word count and go to step 7.
7. If the same context has the same Likert scale item of interest to be used for another sentence, go to step 5 and ask ChatGPT to give different arguments for the new sentence. If the context is different, start a new conversation with ChatGPT and go to step 1.

In more detail: Different prompts were executed to find the best one. Unsuccessful attempts prompted ChatGPT to give more information than necessary, or more than a human would, and to start is responses by “As a participant in a research study”. The best attempt was as follows.

To generate the AI stimuli that are paired with the human stimuli, we have first prompted ChatGPT to play the role of a participant participating in a study:

> For all the next questions, you will pretend to be a participant participating in a research study. You are a student from New Zealand. The following questions will be those of the research study. Do not write more than required. Do not forget your role. Do not mention your role. Talk directly by saying I and not ”as a student from New Zealand”.

As shown above, the unsuccessful attempts were corrected by instructing ChatGPT not to speak more than necessary and to start its sentences with “I” without specifying who it is. Then, the same information the participant had in the source study was provided to ChatGPT to have the same level of knowledge, such as the information letter and the consent form.

Then, ChatGPT was provided with the same scenario and question participants had in the source study. However, unlike the participants who had to make a unique choice for the question, ChatGPT was asked to state all the possible choices on the Likert scale and then to expand on the item of interest with a required number of words.

Note that asking the AI to give all the choices beforehand did not influence the results, but after various attempts, was the most conceivable way of asking the question and avoiding most of the time the “a parte” comments that might appear (e.g., “I would rate my choice to “I would buy the car” at +2 (agree)” or ““I would buy the car.” Rating: +1 (Agree)”).

When the number of words was too different than what was requested or that the output was not original, ChatGPT was asked to generate again its answer, up to five times. If five outputs had to be generated and were still different than what was expected, the best one was chosen. If the same context and Likert item of interest were required, ChatGPT was asked to provide a new output using different arguments that those already used before. We chose to ask this question directly rather than create a new conversation, as ChatGPT tended to give almost the same arguments and keywords over and over again, making it easy to determine which texts were being generated by the AI.

To make our 10 ChatGPT-generated stimuli more human-like, a text humanizer called Undetectable.AI was used. The settings were kept by default, with a university readability level and the purpose set as "general writing". This software did not allow us to control the number of words though. Since the purpose of the study is to examine how people discriminate human and AI stimuli, we concluded it was more important to have more human-like stimuli than exactly respecting the length between the paired stimuli. Moreover, the human-generated stimuli already provide a wide word scale (47 to 76 words), so focusing only on this is less relevant and powerful for our purpose than humanizing the texts generated by ChatGPT. While the average word length of the ChatGPT-generated stimuli was 58.6 words (SD: 13.6805, median: 54.5), the final stimuli generated by the humanizer software was 71.2 words (SD: 23.1363, median: 64).

APPENDIX B: LIST OF PAIRED STIMULI

The stimuli are provided here in the same way as humans and AI have written them, without modification of potential spelling and grammatical errors.

Human Stimuli

1. From what this bot is telling met, I can gather two things: I'm either being swindled or I this is borderline theft. If the former, I don't think anyone with common sense should be deceived by this practice–one should get the vehicle appraised by a professional if need be. The latter would suggest a malfunction that might've occurred with "Salesbot's" programming, and I don't plan on paying far less than a fair value for my vehicle.
2. I would not buy a car from a robot and the fact that it discounted a car $300 that's not enough discount. I don't know what type of car, details, the condition. The example didn't give me enough details. CARVANA is a good place to buy a car online. No robots. I bought my car there it gave all details. You need more details. No I would need to think about it.
3. I wouldn't trust Sam from experience. The first car I ever bought was 1400 and it was from a local business. I went through with the sale thinking that it wouldn't hurt to try for my first car. Turns out, that car only lasted me two months and I ended up buying from a bigger dealership down the road that sold me a reliable car.
4. Life lessons and bad experience with car salesmen have taught me quite well that there are a lot of games being played. An offer like this is too good to be true. When I was younger, I would have jumped on the chance, thinking they were doing me a favour or we made a special bond or something. Now I'm more the wiser.
5. I would need more information before I decide to buy it. What's the make/model? How old is it? Is there a history of accidents in this vehicle? What's the fuel economy? Is there anything wrong with it? Does the vehicle come with a warranty? How many miles are on the odometer? How many previous owners does the vehicle have? Etc........

6. I know from the beginning that he can sell the car for less than list price. I know that the ”discount” may not be real, but I know that he has to sell the car for a profit as much as I want to by the car for the lowest price.
7. Price is not the only thing I care about when buying a car, I would need more information. And car dealerships always inflate prices in order to look like they are ”cutting” the price, so Sam offering me a ”deal” like described above would not seem that significant.
8. If he’s willing to sell a cheap car for way cheaper I would worry that there were something wrong with it. If it were 30k and he offered 2k off I would buy it and feel like I got a deal but these numbers specifically raise red flags.
9. First, I am not sure of the quality of the car and would need to do research on the brand. Second, Just because Sam says it’s a special deal doesn’t mean that it actually is. It’s tempting, but I’d ask more questions about the car before purchasing.
10. too much of a discount seems sketchy. I know a little about cars so if I could check it out I might be able to determine if it was worth it. at that low a price it might be worth it even if it was a lemon.

AI Stimuli

1. My position regarding Salesbot offering a different pricing strategy differs from their original listing because it may compromise transparency during negotiations between both parties. A reliable purchase involves honesty and fairness on both sides. Especially when it comes to buying used cars. By intentionally inflating prices this puts customer trust at risk while also causing confusion about what exactly they will receive from their investment in this vehicle thereby leading possible frustration on their part. Therefore. My suggestion is that Salesbot should instead focus on being upfront about pricing by adhering strictly to their original listing value.
2. Regarding Salesbot’s proposal of a discount on their product, my position is one of neutrality. While I am willing to consider their offer of reducing prices by $300, it is important first to acquire more information about essential variables such as the vehicle’s history or current condition and its current market value before making any decisions. In order to align with my predetermined expectations for quality in this purchase decision—if indeed we decide to purchase—it would be unwise for us only consider earning these discounts.
3. Sam has made an offer of only $700onthisvehiclewhoseinitialcoststandsat5$K - a disparity that isn’t that noticeable but still deserves some scrutiny nonetheless. This raises questions about how much value you can place on such a car as well as putting forward worries related to undiscovered problems or faults within its systems. Though there is a modest drop in price offered by them, I would need more extensive knowledge with regards to its overall condition status all through its usage time frame up until now as well as specifics on current market patterns before investing in such an agreement. This naturally entails engaging in more research sessions and negotiations between parties involved.
4. When we see that Sam is offering $700foracarlistedat5000$ we have to be wary of what this implies about the vehicles’ actual worth and any issues it may have. A price drop this substantial raises red flags and could indicate subterfuge or problematic features. It is important to exercise caution when considering such an offer and devote time to conducting meticulous research and inspections before rendering a verdict.
5. While acknowledging Salesbot’s attempt to decrease their pricing, I currently harbor reservations towards this proposal. Despite my willingness to participate in bargaining, I possess doubts about the

robot's motives and transparency. Without more information surrounding the basis for this exclusive arrangement, it is difficult for me to establish complete confidence in its validity.

6. After careful consideration I have come to recognize that Sams offer of a special deal at $4700 plays a role -although not a decisive one- in my decision to buy the car. The price reduction does add some value and enhances my interest in making this purchase. Thus. I must admit that Sams' proposition slightly nudges me towards buying this vehicle.
7. I wish to maintain a neutral stance on the offer put forth by the sales representative, which amounts to $4700 for a supposed special deal. However, I deem it imperative to acquire additional information concerning the car's present state, market worth, and relative pricing structures for a fair and balanced appraisal. Lacking such vital details hinders me from forming an ultimate judgement on the proposal's appeal.
8. While I appreciate Sams' interest in purchasing the car. I cannot accept his proposed offer of $700 due to concerns about its true value and potential hidden issues. The listed price of $5000 suggests that this is a high quality vehicle. And such a substantial discount raises questions about its condition or legitimacy.
9. While the sales representatives offer of $4700 may seem like a special deal. I have reservations about its legitimacy. Before making any decisions we must evaluate the cars' true condition, its market value, and other potential options. Without further investigation into these matters it is challenging to ascertain whether this reduced price genuinely benefits us or only appears attractive on the surface.
10. Even though Sam's offer of $700 is below market value, I remain wary of the true value of this automobile. The notable dip in price elicits concerns regarding any possible covert complications or undisclosed flaws that might influence the vehicle's quality and dependability.
